# Vision-Based Automated Inspection of Box Meals for Food Portion Defects and Foreign Object Detection

**DOI:** 10.3390/s26175636

**Published:** 2026-09-04

**Authors:** Hong-Dar Lin, Guan-Ming Chen, Chou-Hsien Lin

**Affiliations:** 1 Department of Industrial Engineering and Management, Chaoyang University of Technology, Taichung 413310, Taiwan; s10515610@cyut.edu.tw; 2 Department of Civil, Architectural and Environmental Engineering, The University of Texas at Austin, Austin, TX 78712-0273, USA; chslin@utexas.edu

**Keywords:** machine vision, lunch-box inspection, deep neural network, superpixel segmentation, food portion estimation, foreign object detection

## Abstract

Automated inspection of prepared meals is important for improving food safety, quality assurance, and production efficiency. However, vision-based inspection remains challenging because boxed meals contain multiple adjacent food items with irregular shapes, varying portion sizes, and visually similar appearances, while oily surfaces may introduce specular reflections that degrade image quality. This study presents a vision-based framework for multi-object recognition and quantitative defect analysis in complex meal images, with Chinese-style lunch boxes used as representative test samples. The framework integrates region-of-interest (ROI) extraction, fixed-grid regional feature representation, deep neural network (DNN) classification, flood-filling post-processing, and empirical food-quantity thresholds. The lunch-box ROI is first extracted using the Hough transform, followed by median filtering to suppress reflection noise. The ROI is partitioned into 6 × 6 regions, from which the mean and standard deviation of RGB, HSV, and CIE Lab* color components are extracted and classified using a DNN. Flood filling is subsequently applied to refine the classification results, and category-specific empirical thresholds are used to identify missing food items and insufficient portions. Foreign objects are detected as an additional abnormal category. Under the evaluated experimental conditions, the proposed framework achieved an overall image classification rate (CR) of 96.45%, a defective-image detection rate (1−β) of 97.76%, a normal-image false alarm rate (α) of 5.34%, and a defective-image misclassification rate (γ) of 0.82%. Sensitivity experiments involving illumination variation, two lunch-box configurations with different food compositions, and conveyor-based image acquisition further demonstrated the feasibility and stability of the framework under the evaluated laboratory and prototype conditions. These findings support the feasibility of the proposed lightweight framework for vision-based quality inspection of representative boxed meals, while broader validation across meal types, contaminants, and industrial production environments remains necessary.

## 1. Introduction

Vision-based inspection of prepared meals represents a challenging multi-object recognition and quantitative analysis problem because multiple food categories coexist within a confined scene, often exhibiting irregular boundaries, partial occlusions, specular reflections from oily surfaces, and substantial intra-class appearance variations [1,2]. These characteristics significantly reduce the effectiveness of conventional image segmentation and object classification methods. Consequently, developing robust feature representations and classification strategies capable of accurately recognizing adjacent food components under such conditions remains an important research problem in computer vision [3,4,5]. Automated lunch-box inspection provides a representative application scenario because it requires simultaneous food recognition, quantitative portion evaluation, and foreign-object detection under practical production conditions.

The rapid growth of ready-to-eat meals and centralized food manufacturing has substantially increased the demand for reliable automated food quality inspection systems. In commercial meal production, boxed meals must contain not only the correct combination of food items but also appropriate serving portions and be free from foreign-object contamination. Conventional inspection relies primarily on human inspectors and is therefore labor-intensive, time-consuming, and susceptible to fatigue, subjective judgment, and inconsistent decision-making. These limitations become increasingly significant in high-throughput production environments, where inspection accuracy directly influences production efficiency, product quality, and consumer confidence. As a result, machine vision has become an attractive solution for improving inspection consistency, efficiency, and quality assurance in intelligent food manufacturing [1,2,3].

Despite recent advances in computer vision and artificial intelligence, automated inspection of prepared meals remains considerably more challenging than the inspection of individual food products [4,5]. Chinese-style lunch boxes typically contain multiple adjacent food categories with irregular shapes, partial overlap, and substantial variations in color, texture, and portion size. Furthermore, oily food surfaces generate specular reflections, while visually similar food items often exhibit overlapping color distributions, increasing the difficulty of accurate segmentation and classification. From a quality-control perspective, manufacturers require not only reliable food recognition but also quantitative evaluation of food portions and detection of foreign-object contamination. These combined requirements make comprehensive lunch-box inspection a challenging computer vision task.

To address these challenges, this study proposes a vision-based inspection framework for automated quality assessment of representative Chinese-style lunch boxes and evaluates its performance using two commercially available Taiwan Railway Administration (TRA) lunch-box products. The proposed framework integrates region-of-interest (ROI) extraction using the Hough Transform, median filtering for reflection suppression, superpixel-based color feature extraction, deep neural network (DNN) classification, flood-filling post-processing, and empirical food-quantity thresholding into a unified inspection system. The framework simultaneously performs food recognition, food-portion evaluation, and foreign-object detection, providing an efficient and practical solution for automated meal inspection.

The principal contribution of this study is the development of a lightweight vision-based inspection framework that integrates multiple established computer vision techniques for simultaneous food recognition, quantitative food-portion evaluation, and foreign-object detection, rather than proposing fundamentally new image processing algorithms. It can be divided into four parts. First, an integrated vision-based inspection framework is developed to simultaneously perform multi-class food recognition, quantitative food-portion evaluation, and foreign-object detection for Chinese-style lunch boxes. Second, a lightweight superpixel-based DNN classification strategy combined with flood-filling post-processing is proposed to improve segmentation continuity and classification feasibility for adjacent and partially overlapping food items. Third, an empirical food-quantity thresholding method is introduced to distinguish among normal, insufficient, and missing food portions, extending conventional food classification toward automated quality assessment. Finally, comprehensive experiments, including parameter optimization, classifier comparison, varying illumination conditions, two representative lunch-box geometries with different food compositions, and dynamic conveyor-belt inspection, were conducted to evaluate the feasibility of the proposed framework.

The remainder of this paper is organized as follows. Section 2 reviews recent studies on machine vision, deep learning, food recognition, food portion estimation, and foreign-object detection. Section 3 presents the proposed inspection framework and methodology. Section 4 reports the experimental results, feasibility evaluation, and performance analysis. Finally, Section 5 concludes the paper and discusses future research directions.

## 2. Related Work

Recent advances in machine vision, artificial intelligence, and deep learning have significantly accelerated the development of automated food inspection technologies. Image-based inspection systems provide rapid, objective, and non-destructive evaluation while reducing labor costs and human subjectivity. Consequently, extensive research has been devoted to food quality inspection, food recognition, image segmentation, food portion estimation, and foreign-object detection. This section reviews representative studies in these research areas and identifies the remaining challenges that motivate the proposed vision-based lunch-box inspection framework.

### 2.1. Machine Vision for Automated Food Inspection

Machine vision has become a key enabling technology for intelligent food manufacturing because it provides rapid, objective, and non-contact inspection of food products. Compared with manual inspection, machine vision systems offer higher inspection speed, improved repeatability, and greater consistency while substantially reducing labor costs and operator-dependent variability [1,2,3,4,5]. These systems have been widely applied to quality grading, defect detection, sorting, contamination inspection, and process monitoring in agricultural and food industries. Comprehensive reviews by Brosnan and Sun [1,2], Patel et al. [3], Sun [4], and Wu and Sun [5] demonstrate that machine vision effectively evaluates food appearance, color, size, shape, texture, and surface defects and has evolved into real-time industrial inspection with advances in imaging sensors, computational hardware, and artificial intelligence [6,7,8].

Despite these developments, most machine vision applications focus on individual food products under relatively simple imaging conditions. Prepared meals present considerably greater challenges because multiple food items are arranged within confined containers with irregular shapes, adjacent boundaries, partial overlap, and varying appearances after cooking. Moreover, specular reflections from oily food surfaces and visually similar food categories further complicate accurate segmentation and classification. These challenges require more robust vision algorithms capable of simultaneously handling multi-object recognition, irregular object boundaries, partial occlusion, and complex illumination conditions encountered in practical meal inspection.

### 2.2. Deep Learning for Food Recognition and Image Segmentation

Deep learning has fundamentally transformed food image analysis by enabling automatic hierarchical feature learning directly from raw images. Compared with conventional handcrafted descriptors, convolutional neural networks (CNNs) provide substantially improved recognition accuracy and robustness for food classification [6,7,8,9,10,11,12,13]. Representative studies such as DeepFood [9] and FoodNet [10] demonstrated the superiority of deep learning for food recognition, while publicly available datasets and benchmark studies [14,15,16,17] have accelerated algorithm development. More recently, transfer learning has further improved classification performance while reducing training data requirements.

Accurate image segmentation is equally important because reliable localization directly affects food recognition and subsequent quantitative analysis. Matsuda et al. [18] proposed candidate-region detection for multiple-food recognition, whereas Zhu et al. [19] combined segmentation and classification within a unified framework. Recent semantic and instance segmentation architectures, including U-Net, DeepLabV3+, Mask R-CNN, and YOLO-based segmentation models [7,11,12,13,20,21], have demonstrated excellent performance in pixel-level object recognition. However, these methods generally require extensive pixel-level annotations and considerable computational resources, limiting their applicability to real-time industrial inspection. Furthermore, adjacent food items, irregular boundaries, partial occlusion, and specular reflections remain major challenges in prepared meal analysis.

Recent studies have also investigated statistical representations of pixel distributions for image classification. For example, Tan et al. [22] proposed a differentiable histogram module that learns discriminative pixel-distribution features within a neural network. Similarly, the proposed framework employs lightweight statistical representations by extracting superpixel-based color statistics from the RGB, HSV, and CIE Lab* color spaces, providing an efficient feature representation for food recognition and subsequent quality inspection.

### 2.3. Food Portion Estimation and Foreign Object Detection

In addition to food recognition, automated meal inspection requires quantitative evaluation of food portions and reliable detection of foreign objects. Existing food portion estimation methods have been primarily developed for dietary assessment and nutritional analysis using geometric measurements, depth sensing, three-dimensional reconstruction, or multi-view imaging [23,24,25,26,27]. Representative approaches include smartphone-based estimation without fiducial markers [24], monocular 3D reconstruction [25], and GAN-based food energy estimation [26]. Although these techniques achieve satisfactory estimation accuracy, many require additional hardware or complex reconstruction algorithms and are therefore less suitable for real-time industrial inspection.

Foreign-object detection has traditionally relied on metal detectors, X-ray imaging, hyperspectral imaging, and other non-destructive sensing technologies [28,29]. More recently, machine vision and deep learning have emerged as effective alternatives for detecting food contaminants [30,31]. Nevertheless, foreign-object detection in prepared meals remains particularly challenging because contaminants must be distinguished from multiple food categories exhibiting diverse colors, textures, irregular shapes, and specular reflections. Moreover, existing studies generally investigate food recognition, portion estimation, and contaminant detection separately, with relatively few attempts to integrate these inspection tasks into a single automated framework suitable for practical food production. Consequently, there remains a need for a unified inspection framework capable of simultaneously performing food recognition, quantitative portion evaluation, and foreign-object detection while maintaining computational efficiency suitable for industrial deployment.

### 2.4. Research Gap and Motivation

Recent object detection and instance segmentation models, such as the YOLO family [20,21], have demonstrated remarkable performance in object localization and recognition. However, automated lunch-box inspection requires not only food recognition but also accurate quantitative evaluation of food portions and identification of missing food items and foreign objects. To achieve these objectives, the proposed framework adopts a lightweight superpixel-based representation that provides regional information for food-area measurement while reducing annotation requirements and computational complexity.

Although considerable progress has been achieved in automated food inspection [32,33,34], several important limitations remain. First, existing studies generally address food recognition, food portion estimation, or foreign-object detection independently rather than providing comprehensive inspection of prepared meals. Second, many methods rely on computationally intensive segmentation algorithms or additional imaging hardware that limit real-time industrial deployment. Third, relatively few studies evaluate inspection performance under realistic production conditions involving varying illumination, different lunch-box geometries, diverse food compositions, and conveyor-belt transportation.

Motivated by these limitations, this study proposes a unified vision-based inspection framework for automated lunch-box quality inspection. The proposed framework integrates ROI extraction, superpixel-based feature representation, deep neural network classification, flood-filling post-processing, empirical thresholding for food-portion evaluation, and foreign-object detection into a single inspection system. By combining classical image processing with lightweight deep learning, the proposed approach simultaneously addresses the challenges of multi-object recognition, quantitative food-portion evaluation, and foreign-object detection under practical imaging conditions involving specular reflections, adjacent food items, and varying container geometries.

## 3. Materials and Methods

**Figure 1:**
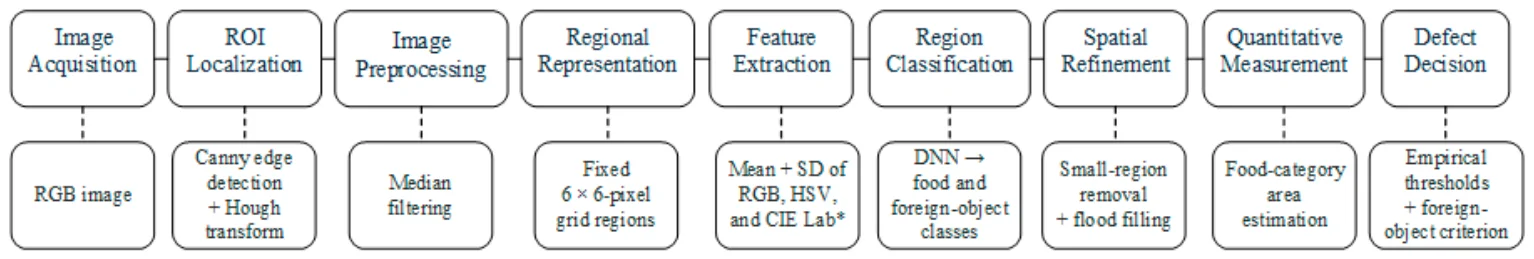
General algorithmic workflow of the proposed vision-based multi-object recognition and quantitative defect-analysis framework (**upper row**) and its implementation for lunch-box inspection (**lower row**).

The proposed method is formulated as a general vision-based framework for multi-object recognition and quantitative defect analysis in complex images, where multiple adjacent or partially overlapping object categories must be identified and quantitatively evaluated for defect determination. As illustrated in Figure 1, the framework comprises nine stages: image acquisition, ROI localization, image preprocessing, regional representation, feature extraction, region classification, spatial refinement, quantitative measurement, and defect decision. This formulation distinguishes the general methodological workflow from the application-specific algorithms used at each stage. In this study, lunch-box quality inspection is used as a representative application, with Canny edge detection and the Hough transform for ROI localization, median filtering for preprocessing, fixed-grid regional representation, statistical RGB/HSV/CIE Lab* features, DNN-based region classification, spatial refinement, and category-specific empirical criteria for quantitative defect determination. The following subsections describe the implementation and experimental evaluation of these stages.

**Figure 2:**
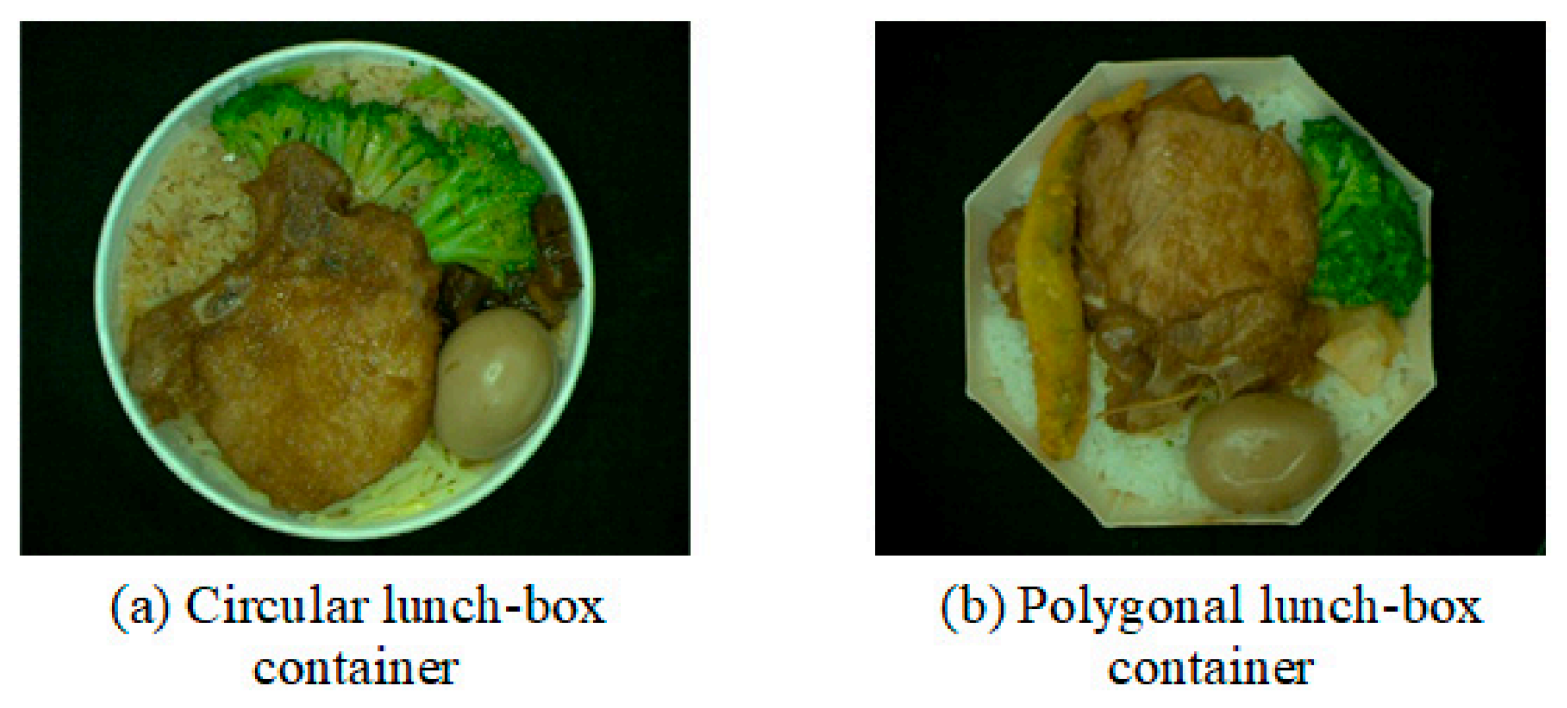
Representative TRA lunch-box containers with different geometries used in this study.

To evaluate the practicality and feasibility of the proposed framework, experiments were conducted using two commercially available TRA lunch-box products with different container geometries. Figure 2 illustrates the representative circular and polygonal lunch-box containers used in this study. Although the container shapes differ, both meal types contain comparable food categories, including rice, meat, vegetables, egg, and side dishes, providing a consistent basis for evaluating food recognition, food-portion estimation, and defect detection. Incorporating different container geometries enables assessment of the proposed framework under practical packaging conditions, where variations in container shape directly influence ROI extraction and subsequent image analysis. The following subsections describe the image acquisition procedure, image preprocessing, feature extraction, food classification, post-processing, and defect detection algorithms.

### 3.1. Image Acquisition and Dataset Preparation

**Figure 3:**
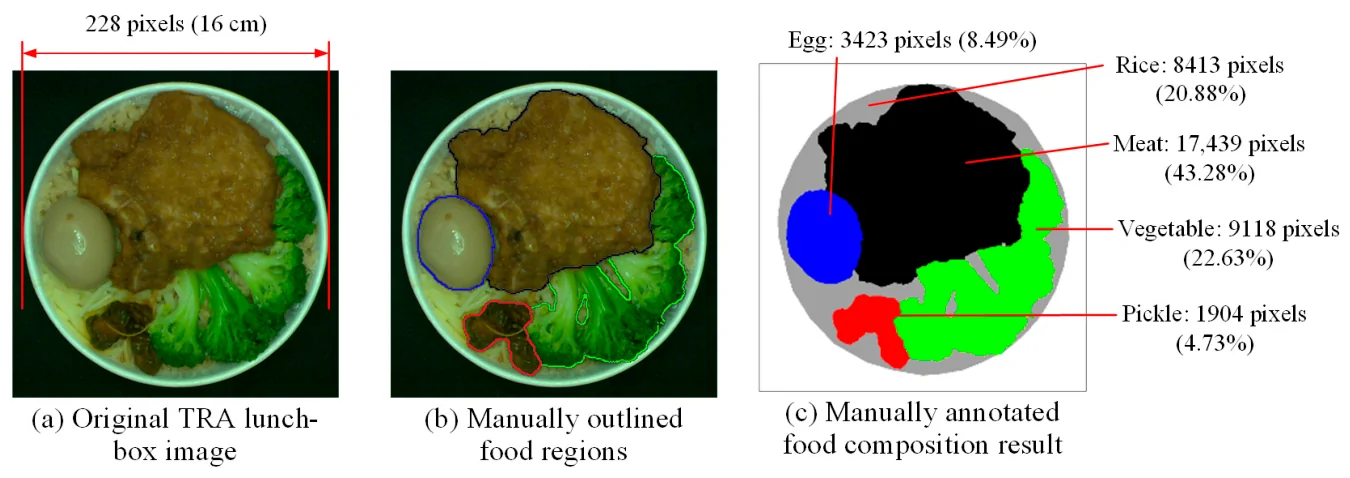
Image acquisition and manual annotation workflow for food region identification and portion quantification.

**Figure 4:**
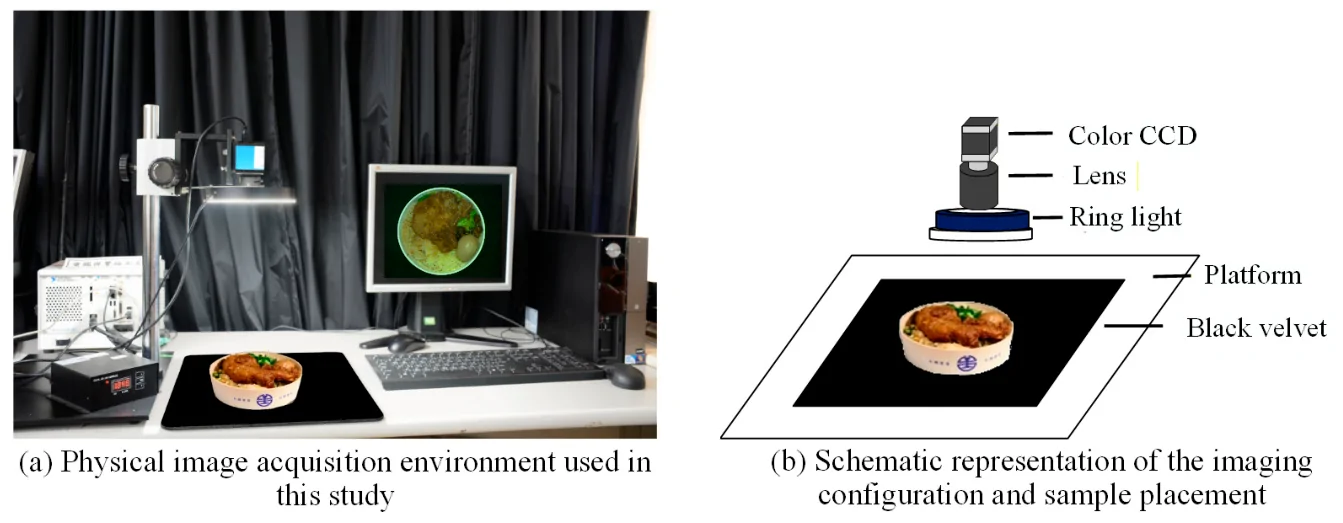
Experimental setup of the lunch-box image acquisition system.

To establish a dataset for food recognition, portion estimation, and foreign-object detection, an image acquisition and annotation procedure was developed, as illustrated in Figure 3 and Figure 4. Each lunch-box image was captured under controlled imaging conditions and manually annotated to identify five food categories, namely rice, meat, vegetables, egg, and pickled side dishes. The annotated food regions were subsequently converted into pixel-level masks, from which the area of each food category was calculated to provide ground-truth information for food classification, portion evaluation, and defect analysis, as shown in Figure 3.

The image acquisition system is illustrated in Figure 4. It consisted of a color CCD camera equipped with a 10 mm lens and a ring light mounted above a fixed imaging platform. A black velvet background was used to suppress background interference and reduce surface reflections, while all lunch-box samples were placed at a fixed position to ensure consistent imaging conditions. This configuration enabled the acquisition of high-quality images for subsequent preprocessing, feature extraction, and food classification.

**Figure 5:**
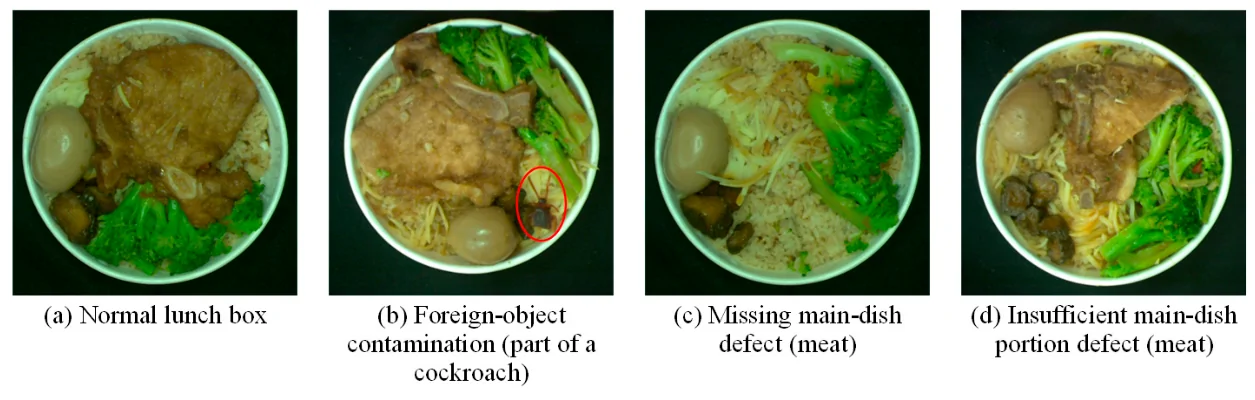
Representative normal and defective lunch-box samples used for system evaluation. The red circle in (**b**) indicates the location of the simulated foreign object (part of a cockroach).

To evaluate the proposed inspection framework under practical manufacturing conditions, both normal and defective lunch-box samples were included in the dataset. As illustrated in Figure 5, the defect categories comprised foreign-object contamination, missing main dishes, and insufficient main-dish portions, together with normal lunch boxes as reference samples. These representative scenarios cover the most common quality defects encountered in commercial lunch-box production and provide a comprehensive basis for evaluating food recognition, portion estimation, and defect detection performance.

### 3.2. Region-of-Interest Extraction

**Figure 6:**
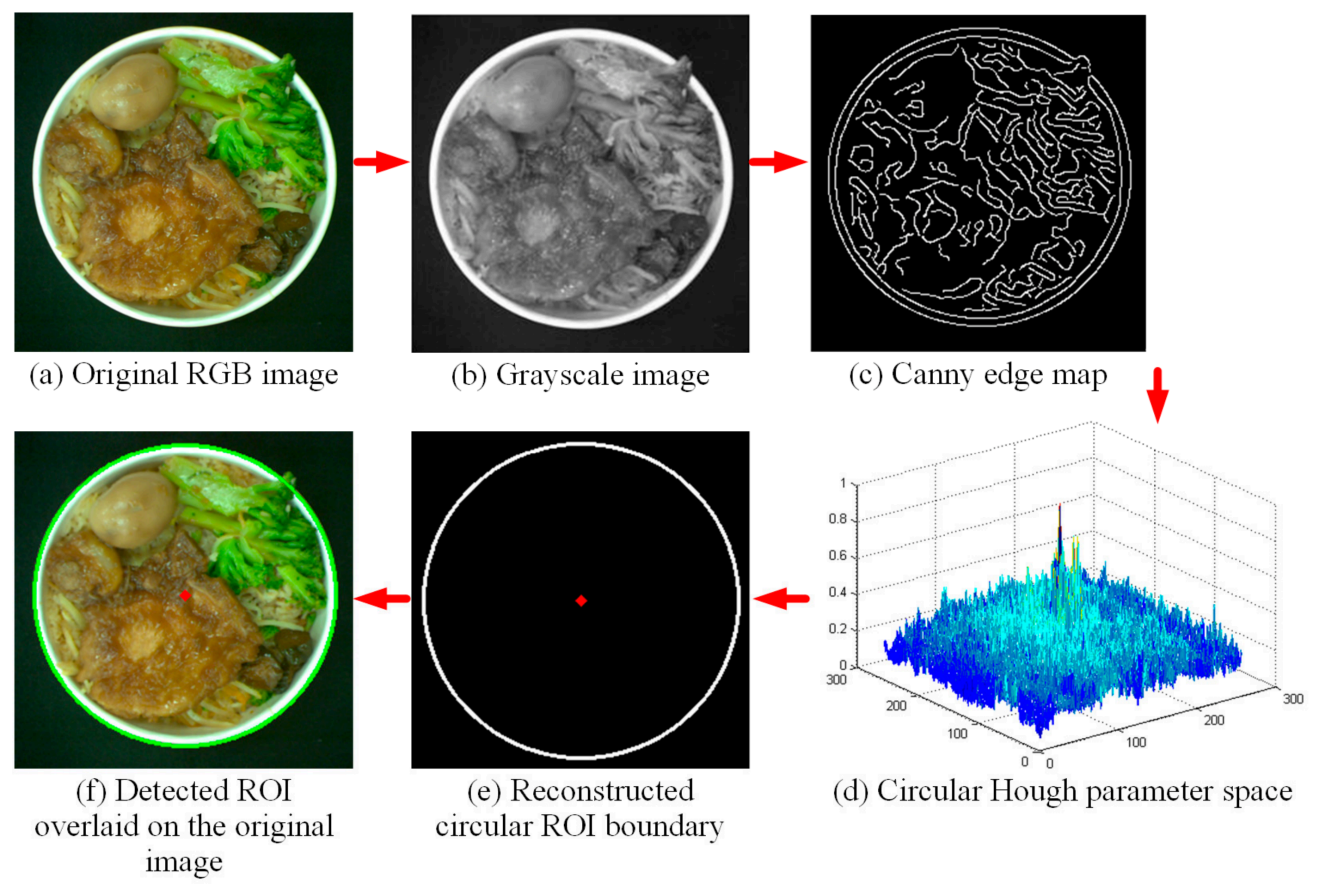
ROI extraction procedure using grayscale conversion, Canny edge detection, and the Circular Hough Transform. Red arrows indicate the processing sequence, the green circle indicates the detected ROI boundary, and the red marker indicates the detected center of the circular ROI.

The lunch-box container was first localized to define the ROI for subsequent food segmentation and analysis. As illustrated in Figure 6, the input RGB image was first converted to a grayscale image to reduce computational complexity while preserving structural information. Canny edge detection [35] was then applied to extract the container boundary, followed by the Circular Hough Transform [36] to identify the circular ROI based on the peak response in the Hough parameter space. The Canny detector provides improved noise suppression, more accurate edge localization, and better edge continuity through Gaussian smoothing, non-maximum suppression, and hysteresis thresholding. These properties produce reliable container boundaries that facilitate robust Hough Transform-based detection of circular and polygonal lunch-box geometries. The extracted edge map serves exclusively as the input to the Hough Transform for container boundary detection, whereas subsequent food recognition is performed using superpixel-based color features and DNN classification.

The detected boundary was subsequently reconstructed and superimposed on the original image to verify the localization result. The extracted ROI effectively removes irrelevant background regions and provides a standardized analysis area for subsequent image preprocessing, feature extraction, food classification, portion estimation, and defect detection. Because the container boundary is determined automatically, the proposed approach is robust to variations in food arrangement and image content while maintaining consistent ROI extraction across lunch-box samples.

### 3.3. Reflection Suppression by Median Filtering

**Figure 7:**
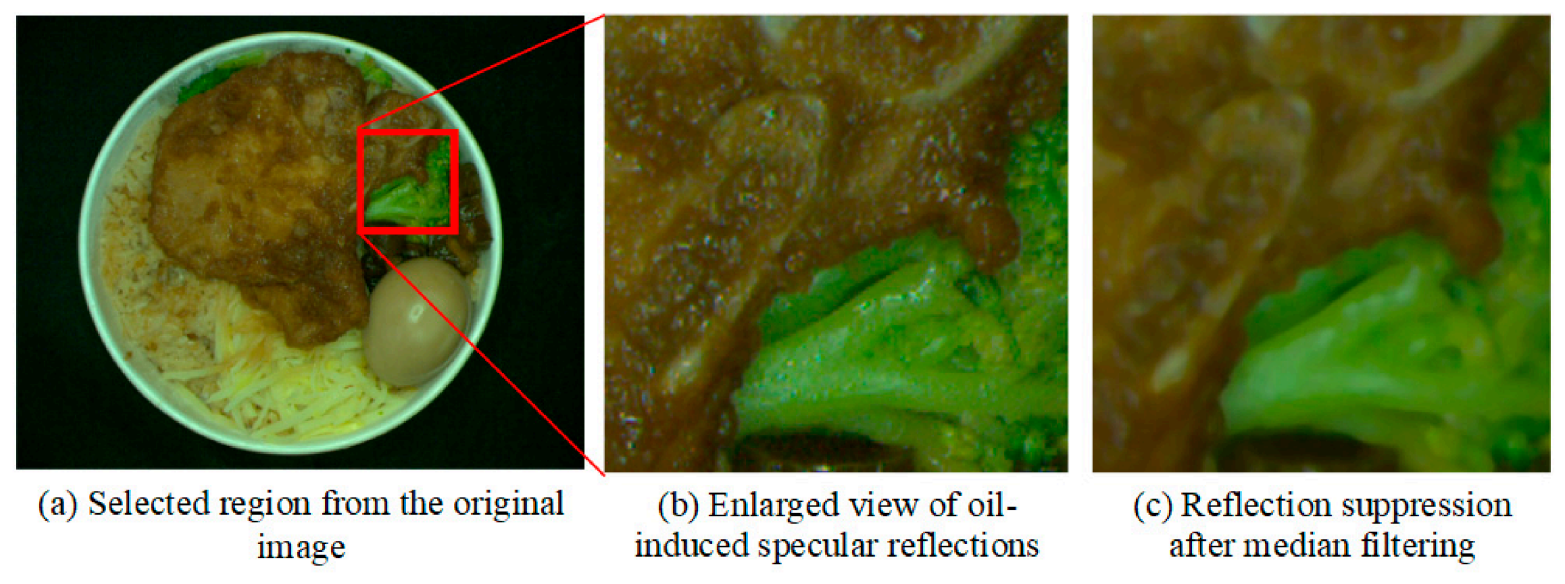
Suppression of oil-induced specular reflections using median filtering.

Lunch-box images were acquired after food preparation, where oily food surfaces frequently produced specular reflections under the ring-light illumination. These reflections introduced local intensity variations and distorted color information, thereby reducing the reliability of color feature extraction and degrading the accuracy of food segmentation and classification. Median filtering suppresses high-frequency specular reflections caused by oily food surfaces while preserving regional structures required for reliable color feature extraction. As illustrated in Figure 7, median filtering effectively reduces high-intensity reflective artifacts while preserving food boundaries and structural details. This preprocessing step improves image smoothness, stabilizes color distributions, and enhances the feasibility of subsequent segmentation, feature extraction, food recognition, and defect detection. Consequently, median filtering provides an effective and computationally efficient solution for reducing oil-induced reflective noise in the proposed lunch-box inspection system.

### 3.4. Color Feature Extraction

Color is one of the most informative visual features for food recognition because different food categories exhibit distinct chromatic characteristics after cooking [5]. To comprehensively characterize food appearance, three complementary color spaces—RGB, HSV, and CIE L*a*b*—were employed for feature extraction. The RGB color space directly represents the intensity of the red, green, and blue channels; HSV separates color information into hue, saturation, and value, providing better perceptual interpretation; and CIE L*a*b* offers perceptual uniformity and effectively represents subtle color differences. By integrating these three color spaces, the proposed framework extracts complementary color information to improve the feasibility of subsequent food classification.

#### 3.4.1. RGB Color Space

**Figure 8:**
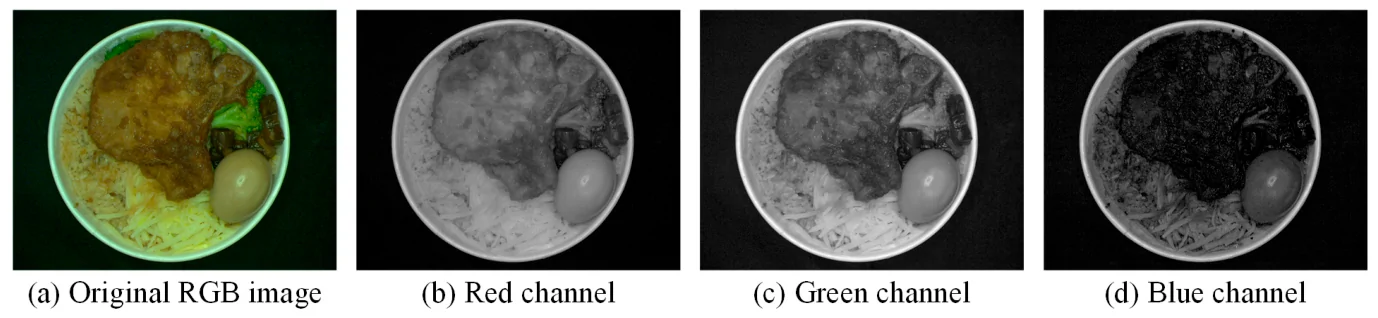
RGB image and its corresponding red, green, and blue channel components.

**Figure 9:**
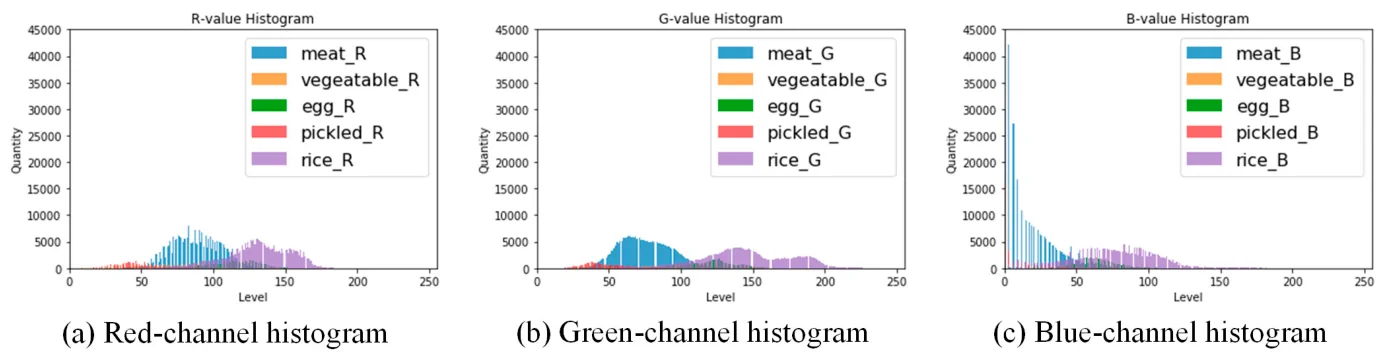
RGB histogram distributions of different food categories.

The RGB color space was first analyzed because it directly corresponds to the image acquisition process and preserves the original color information. As shown in Figure 8, the captured lunch-box image was decomposed into its red, green, and blue channel components to examine the color characteristics of different food categories. The statistical distributions of the RGB channel values are presented in Figure 9. Although each food category exhibits a distinctive color distribution, considerable overlap exists among the RGB histograms, particularly for meat, egg, vegetables, and pickled side dishes. Only rice exhibits relatively better separation in the red channel. These results indicate that RGB features alone are insufficient for reliably distinguishing visually similar food categories. Therefore, complementary color features extracted from the HSV and CIE L*a*b* color spaces are incorporated to enhance feature discrimination and improve classification performance.

#### 3.4.2. HSV Color Space

**Figure 10:**
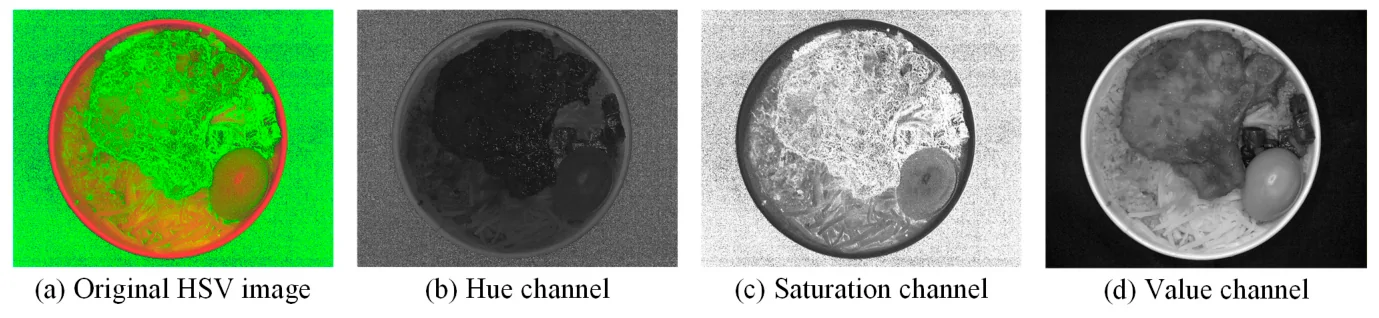
HSV image and its corresponding hue, saturation, and value channel components.

**Figure 11:**
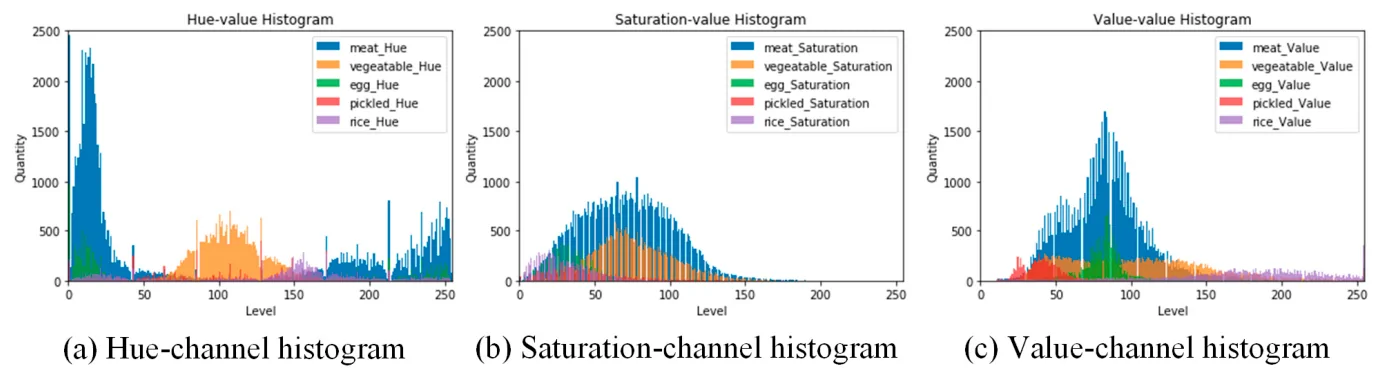
HSV histogram distributions of different food categories.

The HSV (Hue, Saturation, Value) color space was employed because it separates chromaticity, color purity, and brightness, making it less sensitive to illumination variations than the RGB color model. As illustrated in Figure 10, the RGB image was transformed into the HSV color space and decomposed into its hue, saturation, and value components to analyze the color characteristics of different food categories. The corresponding HSV channel histograms are presented in Figure 11. Compared with the RGB color space, HSV provides improved discrimination for several food categories. In particular, rice exhibits a more distinctive distribution in the value channel, while meat and vegetables show greater separation in the hue channel. These results indicate that HSV features effectively complement RGB features by enhancing the discrimination of visually similar food items and improving the feasibility of subsequent food classification.

#### 3.4.3. CIE L*a*b* Color Space

**Figure 12:**
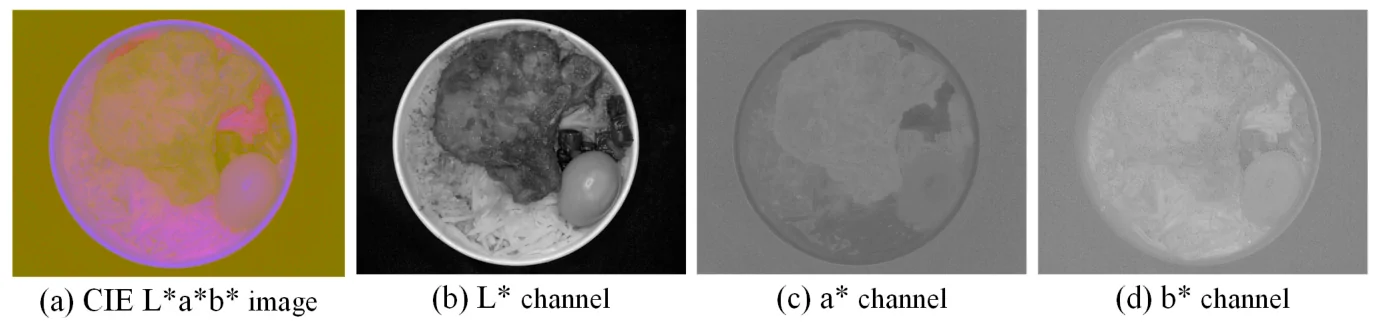
CIE L*a*b* image and its corresponding L*, a*, and b* channel components.

**Figure 13:**
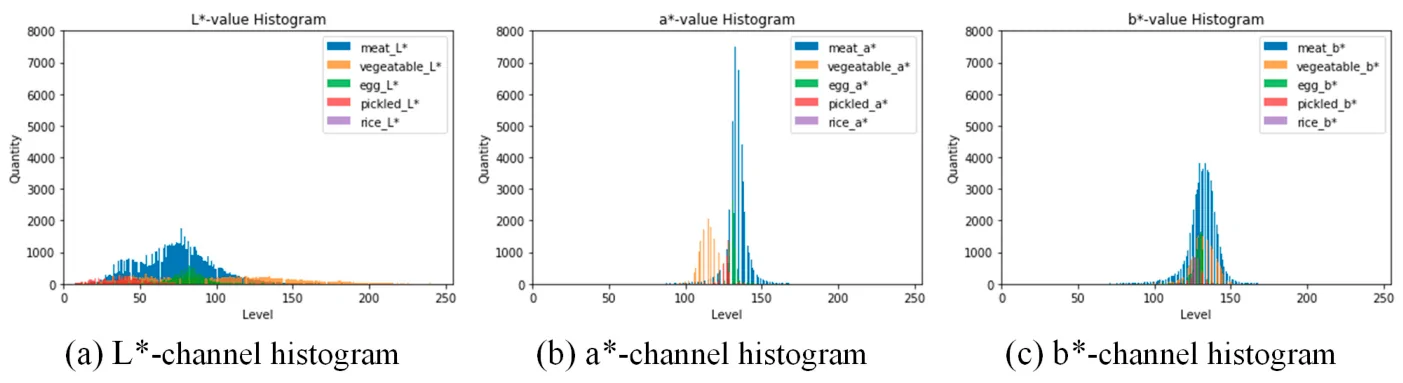
CIE L*a*b* histogram distributions of different food categories.

The CIE L*a*b* color space was employed because it provides a perceptually uniform representation of color by separating lightness (L*) from the chromatic components a* (green–red) and b* (blue–yellow). Compared with RGB and HSV, CIE L*a*b* is less sensitive to illumination variations and is more effective for distinguishing subtle color differences. As illustrated in Figure 12, the lunch-box image was transformed into the CIE L*a*b* color space and decomposed into its L*, a*, and b* channel components for color feature analysis. The corresponding channel histograms are presented in Figure 13. The a* channel provides better separation between meat and vegetables than the RGB and HSV color spaces, indicating that perceptual chromatic information effectively enhances class discrimination. These results demonstrate that CIE L*a*b* features complement the RGB and HSV features by providing additional perceptual color information, thereby improving the feasibility and accuracy of subsequent food classification. The nine channel components obtained from the RGB, HSV, and CIE L*a*b* color spaces were subsequently used to construct the feature vectors for superpixel-based food classification.

### 3.5. Superpixel-Based Feature Representation

**Figure 14:**
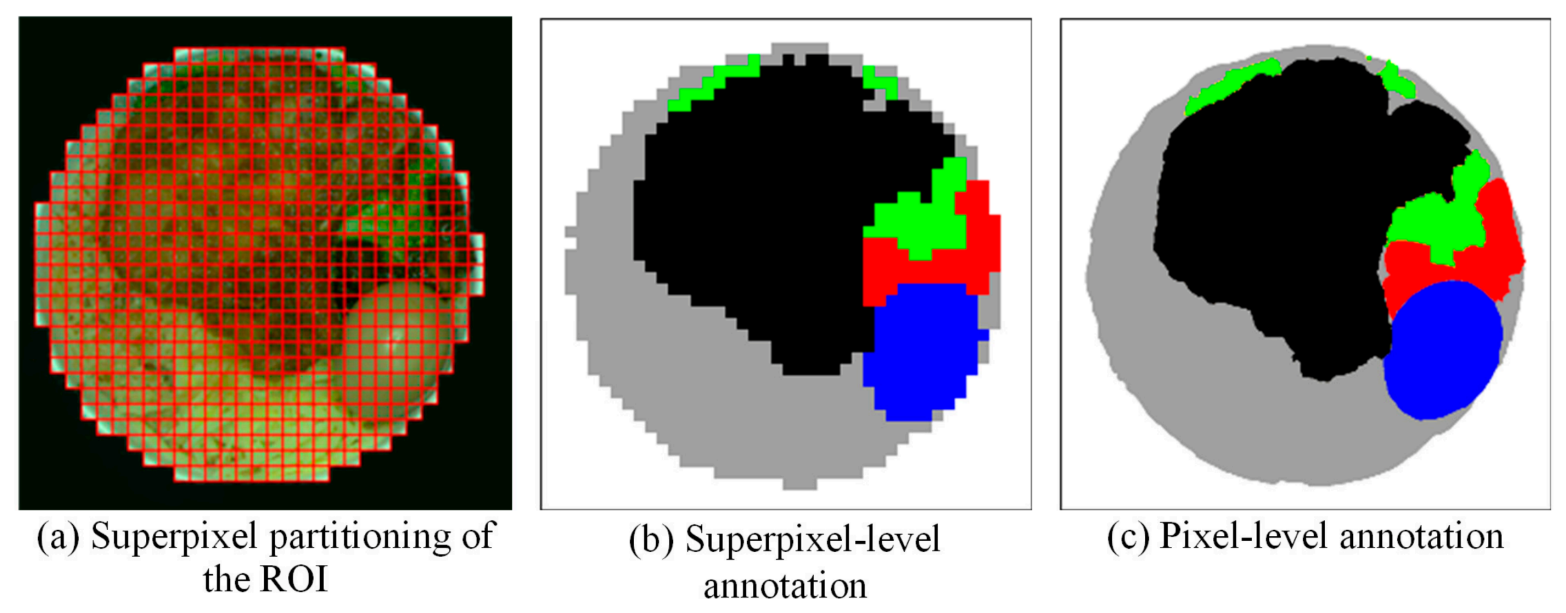
Superpixel partitioning and corresponding superpixel- and pixel-level annotations. The red lines in (**a**) indicate the superpixel boundaries. In (**b**) and (**c**), black, blue, green, red, and gray represent meat, egg, vegetables, pickled side dish, and rice, respectively.

To improve computational efficiency while preserving local image characteristics, the extracted ROI was partitioned into uniform superpixel grids, as illustrated in Figure 14. Superpixels group neighboring pixels into homogeneous regions, providing a compact representation for feature extraction and food classification. Manual annotations were generated at both the superpixel and pixel levels. Superpixel-level annotations were used for classifier training and portion estimation, whereas pixel-level annotations served as the ground truth for evaluating segmentation accuracy.

The size of each superpixel affects both computational efficiency and classification performance. Very small superpixels increase computational cost and reduce the statistical reliability of feature estimation, whereas excessively large superpixels may obscure fine image details and increase boundary misclassification [37]. Based on preliminary experiments, a superpixel size of 6 × 6 pixels was selected as a compromise between spatial resolution and computational efficiency. Consequently, each 256 × 256 image was partitioned into 42 × 42 superpixels (1764 in total), of which approximately 1100 were located within the lunch-box ROI.

Unlike adaptive superpixel algorithms such as SLIC [37], the proposed method employs a fixed-grid partition to provide a computationally efficient and regular regional representation suitable for feature extraction and quantitative food-area analysis. For each superpixel, the mean and standard deviation of the nine color channels (RGB, HSV, and CIE L*a*b*) were calculated, resulting in an 18-dimensional feature vector. All features were subsequently normalized to the range [0, 1] before being input into the DNN classifier.

### 3.6. Deep Neural Network Classification

For food classification, the 18-dimensional superpixel feature vectors were input into a supervised DNN classifier. As summarized in Table 1, the input features consisted of the mean and standard deviation of the RGB, HSV, and CIE L*a*b* color components. The DNN model contained four hidden layers, each with 40 nodes, and produced six output classes: meat, egg, vegetable, pickled side dish, rice, and foreign object.

**Figure 15:**
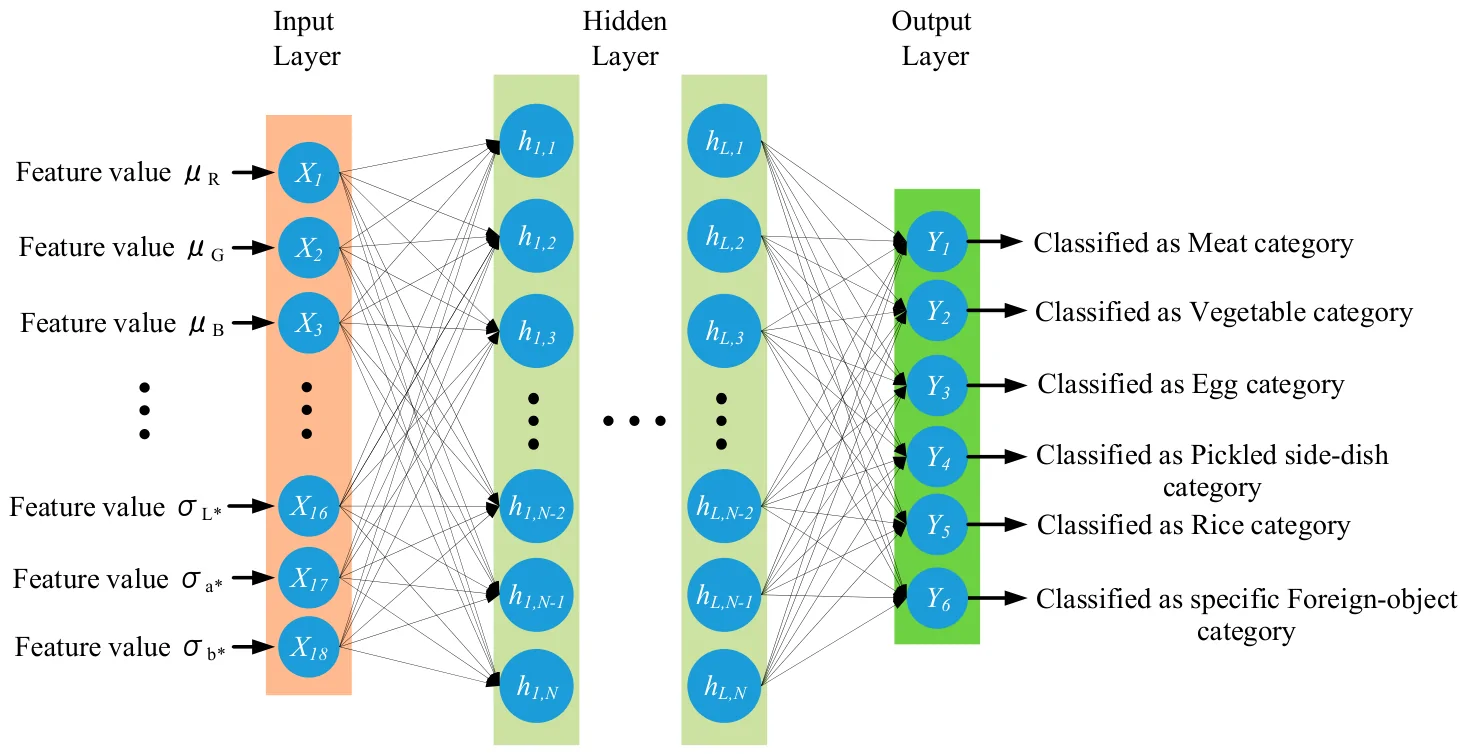
Architecture of the proposed DNN model. The arrows indicate the direction of information flow, and the connecting lines represent fully connected relationships between neurons in adjacent layers. The orange, light-green, and green backgrounds denote the input, hidden, and output layers, respectively, while the blue circles represent neurons.

Before classification, all feature values were normalized to the range [0, 1] using Min–Max normalization to reduce the influence of scale differences among color features and improve training stability. The Rectified Linear Unit (ReLU) was adopted as the activation function in the hidden layers to alleviate the vanishing-gradient problem, while the Softmax function was used in the output layer to generate class probabilities. The schematic architecture of the proposed DNN model is shown in Figure 15.

**Figure 16:**
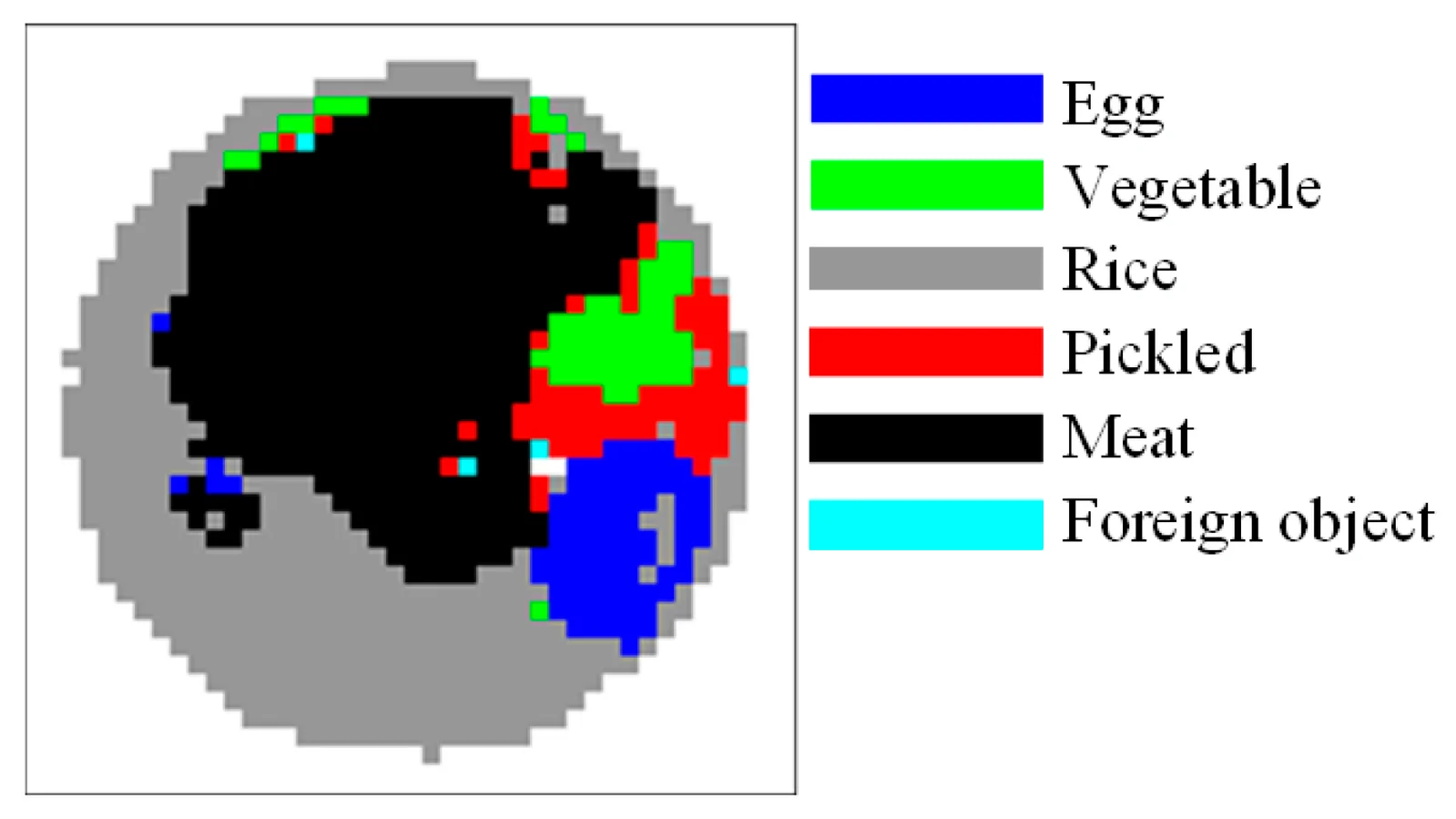
DNN-based classification result of lunch-box contents.

After training, each superpixel was classified into one of the six predefined categories. Figure 16 illustrates an example classification result, where different colors represent different food categories or foreign objects. The classified regions provide the basis for subsequent food portion estimation, defect identification, and foreign-object detection.

### 3.7. Region Refinement by Flood-Filling

**Figure 17:**
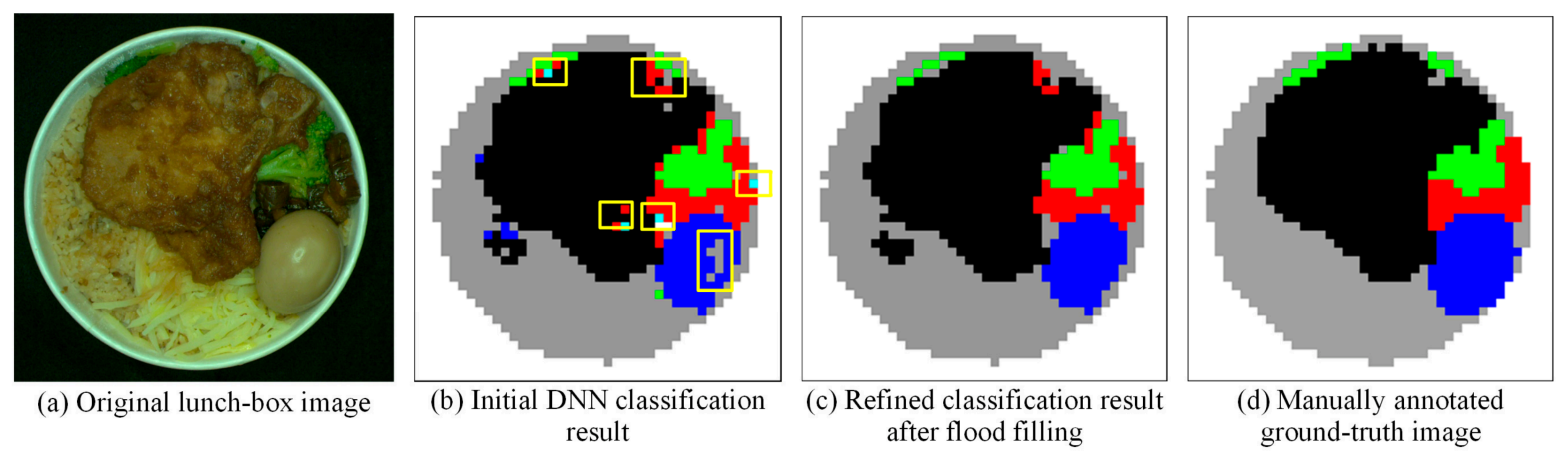
Post-processing of DNN classification results using the flood-filling algorithm. The yellow boxes in (**b**) highlight representative isolated or locally misclassified regions targeted for post-processing. Black, blue, green, red, and gray represent meat, egg, vegetables, pickled side dish, and rice, respectively.

To improve the continuity of the DNN classification results, a post-processing procedure based on the flood-filling algorithm was applied [38]. Flood filling improves region connectivity by correcting isolated superpixel misclassifications introduced by local color ambiguity. As shown in Figure 17, the initial DNN output contained several isolated misclassified superpixels and small noisy regions caused by color similarity and ambiguous boundaries between adjacent food categories. Therefore, connected regions containing fewer than two superpixels were first removed to suppress isolated classification noise. The flood-filling algorithm was then employed to refine the classified regions by filling internal holes and improving regional connectivity [39]. Compared with the original DNN output, the refined result exhibits smoother boundaries and greater consistency with the manually annotated ground truth, thereby providing a more reliable basis for subsequent food portion estimation and defect detection.

**Figure 18:**
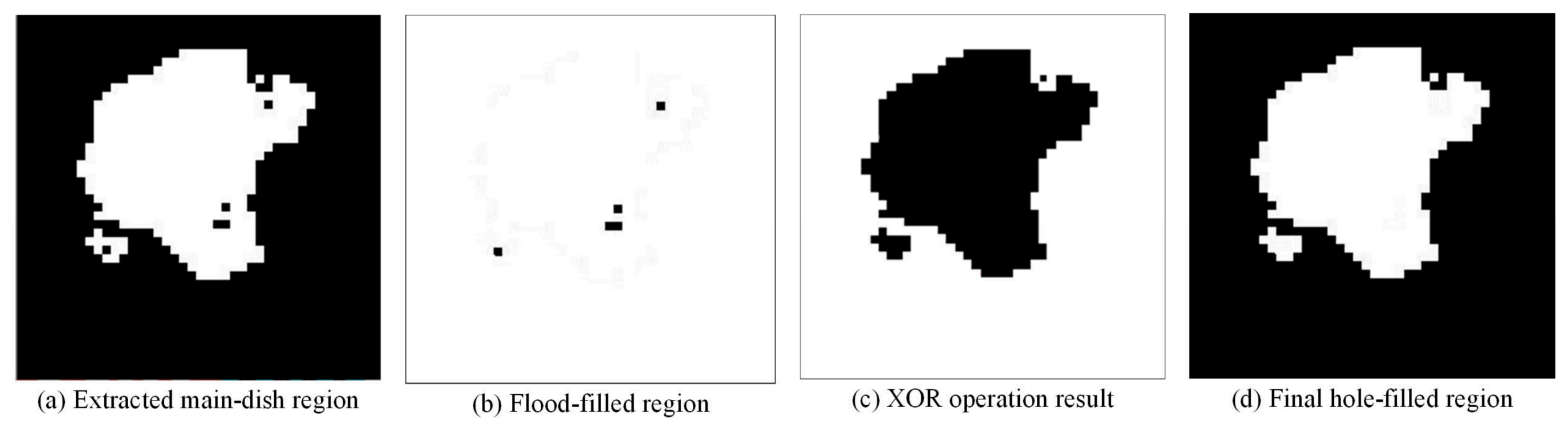
Hole-filling procedure for classified main-dish regions using the flood-filling algorithm.

The hole-filling procedure is illustrated in Figure 18. The classified main-dish regions were first extracted, followed by flood filling to identify enclosed holes. An XOR operation between the original and flood-filled images was subsequently performed to obtain the hole regions, and binary inversion was applied to generate the final filled result. To avoid influencing foreign-object detection, flood filling was applied only to the main-dish categories (meat, egg, and vegetables), whereas the classifications of rice, pickled side dishes, and foreign objects were retained from the original DNN output. This post-processing strategy effectively reduced classification noise, improved region completeness, and enhanced the accuracy of subsequent inspection tasks.

### 3.8. Defect Detection Strategy

Following food classification and post-processing, the proposed system performs automated defect detection to determine whether each lunch box satisfies predefined quality standards. In this study, defects are categorized into two types: (1) food defects, including missing food items and insufficient food portions, and (2) foreign-object contamination. These defects are identified by analyzing the classified food regions and their corresponding spatial distributions.

Food portions are quantitatively evaluated by calculating the area of each classified food category and comparing it with predefined acceptance criteria. Missing and insufficient food portions are detected based on empirical food-quantity thresholding, while foreign-object contamination is identified from the classified foreign-object regions. By integrating food classification, portion evaluation, and foreign-object detection, the proposed framework provides an efficient and comprehensive solution for automated lunch-box quality inspection.

#### 3.8.1. Empirical Interval Measure for Food-Quantity Defects

**Figure 19:**
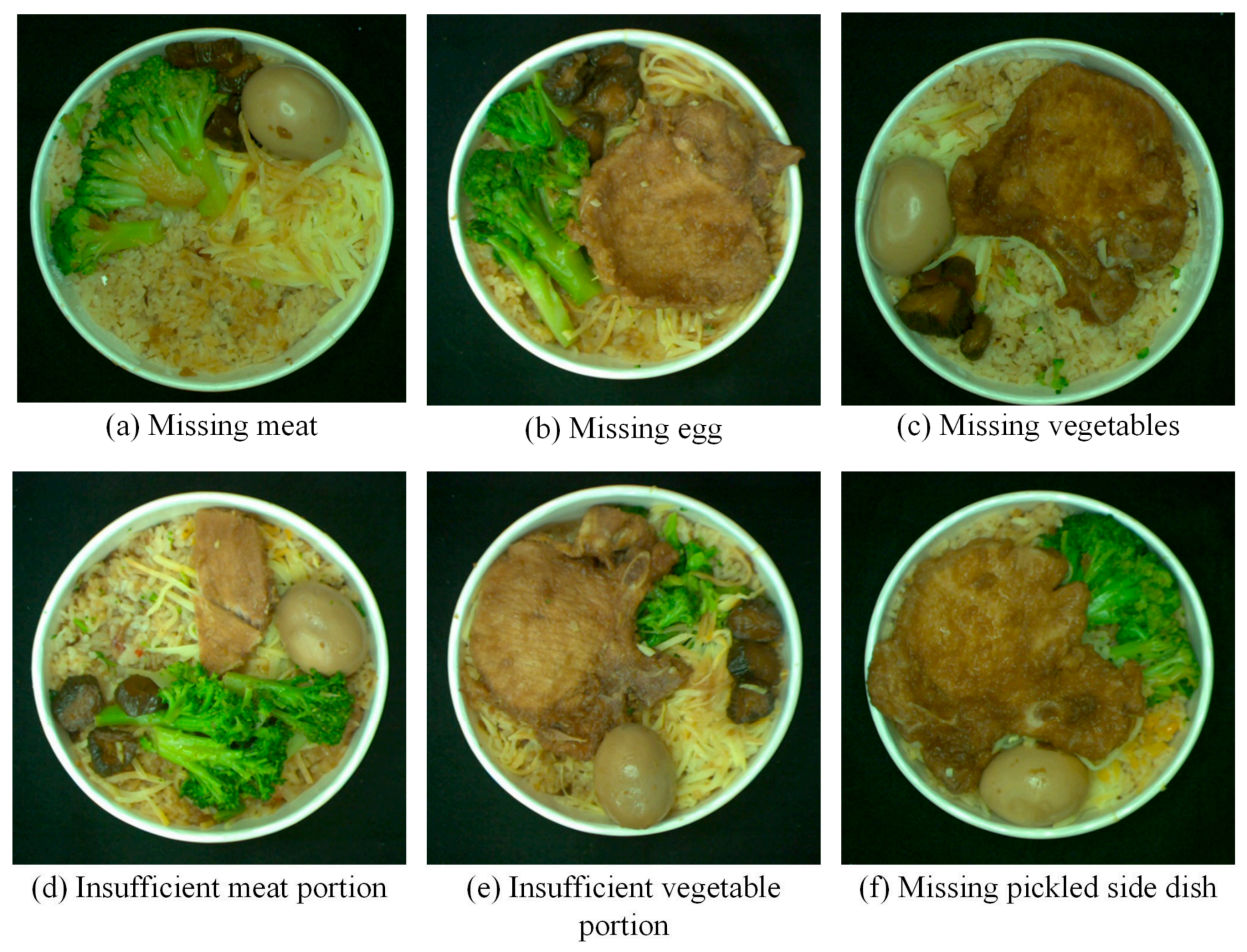
Representative examples of missing and insufficient food portions in different food categories.

The lunch boxes considered in this study contain five food categories: meat (braised pork chop), vegetables (broccoli), egg, pickled side dish (pickled cucumber), and fried rice. As illustrated in Figure 19, the proposed system detects three types of portion abnormalities, including missing food items, insufficient food portions, and missing side dishes. These defects are identified by comparing the measured food area with empirical food-quantity thresholds derived from the training dataset.

To determine the omission and insufficient-portion thresholds for each food category, histograms of the food areas were first generated from the training samples. Decision thresholds were then empirically selected according to the separation between the normal and defective sample distributions. Finally, the selected threshold values were normalized and expressed as multiples of the standard deviation relative to the mean of the normal samples for consistent representation across different food categories. These coefficients do not imply an assumption of Gaussian-distributed food areas.

Let *μ* and *σ* denote the mean and standard deviation of the food area for each category, respectively, and let *X* represent the measured food area in the test image. The parameters *N* and *L* are category-specific threshold coefficients that determine the lower and upper decision boundaries for insufficient-portion detection, respectively. Their values are experimentally determined from the training dataset and vary among food categories, as summarized in Table 2. Accordingly, a food item is classified as having an insufficient portion if

*μ* − *Nσ* < *X* ≤ *μ* − *Lσ*, (1)

and as missing if

*X* ≤ *μ* − *Nσ*. (2)

**Figure 20:**
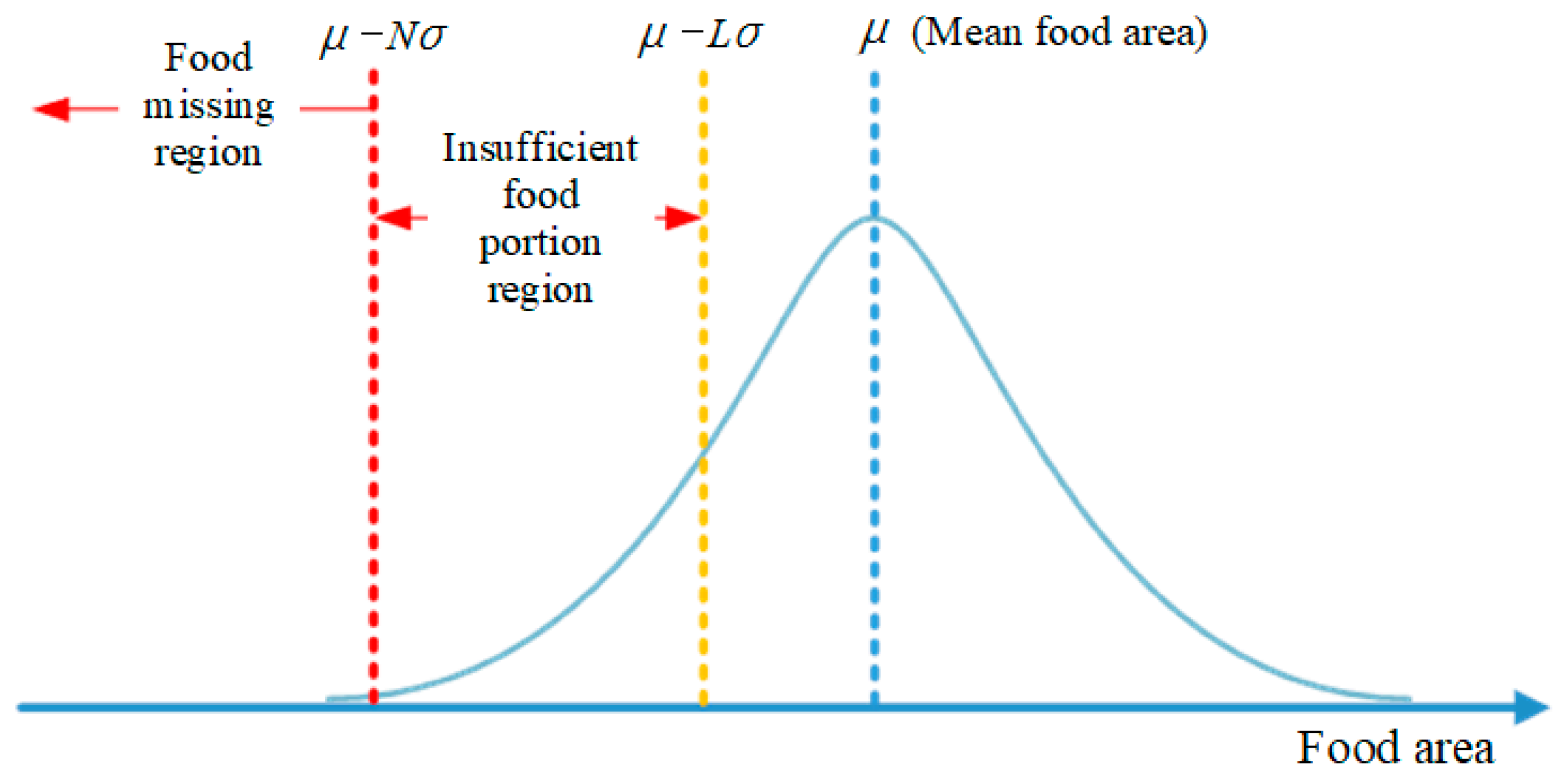
Empirical interval-measure framework for detecting missing and insufficient food portions.

The concept of empirical threshold-based food portion evaluation is illustrated in Figure 20, where food areas are divided into normal, insufficient, and missing regions according to empirical food-quantity thresholds. The corresponding decision thresholds for each food category are summarized in Table 2. Using these criteria, the proposed system automatically detects missing and insufficient food portions for quality inspection.

Food-quantity defects were identified using category-specific empirical thresholds based on the distribution of food areas in the training data. For each food category, the mean (*μ*) and standard deviation (*σ*) of the food area were calculated from the normal training samples. Training samples representing omission and insufficient-portion defects were then used to determine empirical decision boundaries, which were expressed relative to the normal-sample distribution as *μ* − *Nσ* and *μ* − *Lσ*, respectively. Here, σ is used as a normalization measure of the variability in normal food portions; therefore, these expressions should not be interpreted as confidence intervals or Gaussian probability bounds, and no normal-distribution assumption is imposed. Because the area distributions and portion variability differ among food categories, *N* and *L* were determined independently for each applicable category.

The defect definitions were established according to the physical characteristics and serving practices of each food category. Meat and vegetables are continuous food portions whose serving sizes can vary; therefore, both insufficient-portion and missing-food defects were evaluated. In contrast, the braised egg is a discrete food item, with one whole egg served in each lunch box, and its visible area is often partially occluded by the pork chop. Consequently, only the missing-egg defect was considered. Likewise, the pickled side dish exhibits relatively large natural variation in shape and serving quantity, making a reliable insufficient-portion criterion difficult to establish. Therefore, only the missing pickled-side-dish defect was included.

#### 3.8.2. Foreign-Object Detection

**Figure 21:**
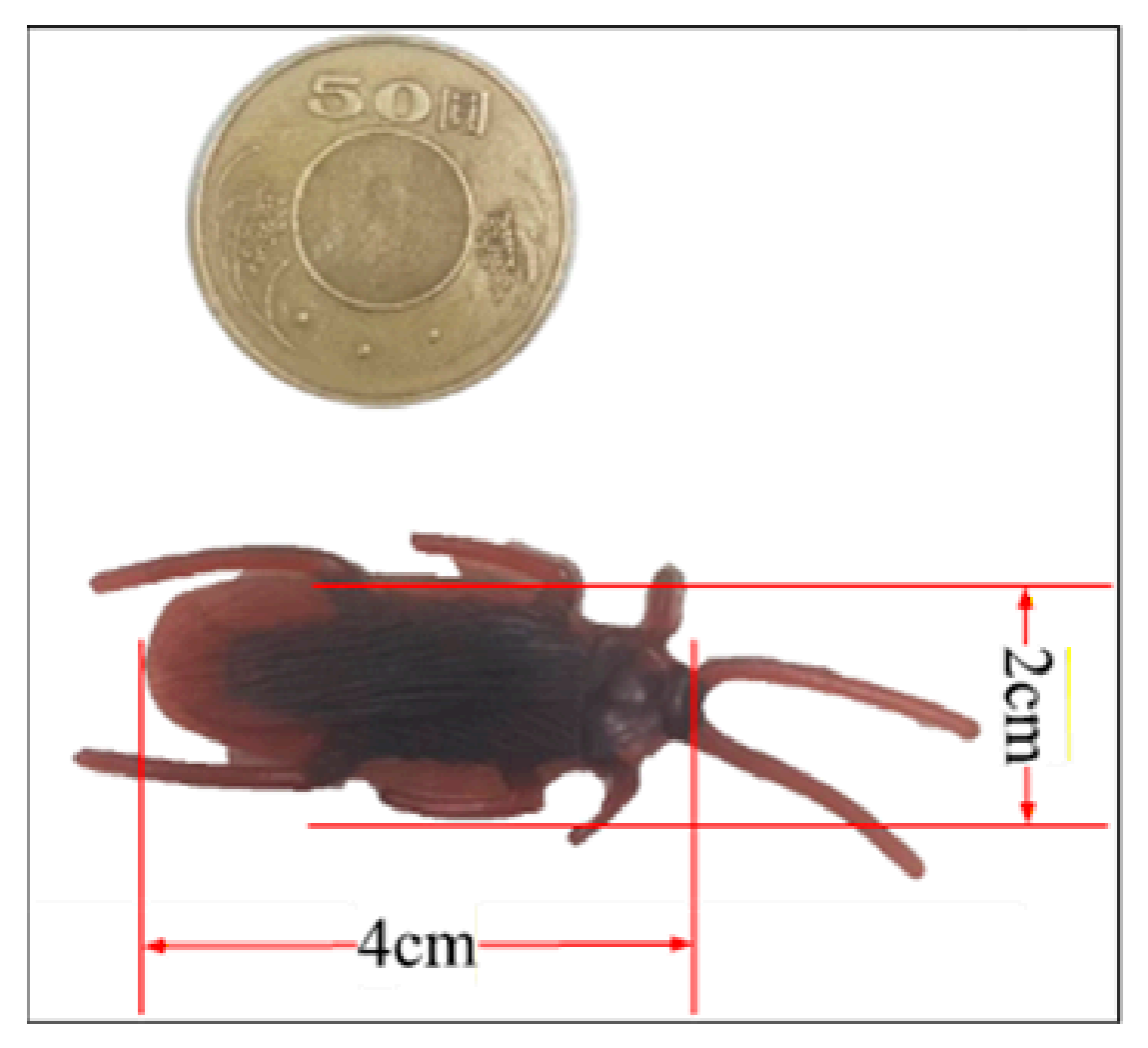
Plastic cockroach model used for foreign-object contamination simulation.

Any object present in a lunch box that is not part of the intended meal is regarded as a foreign object. To simulate contamination under controlled conditions, a plastic cockroach model measuring approximately 4 cm × 2 cm was selected as the representative contaminant because cockroaches are among the most commonly reported foreign objects in commercially prepared lunch boxes. The simulated contaminant is shown in Figure 21.

**Figure 22:**
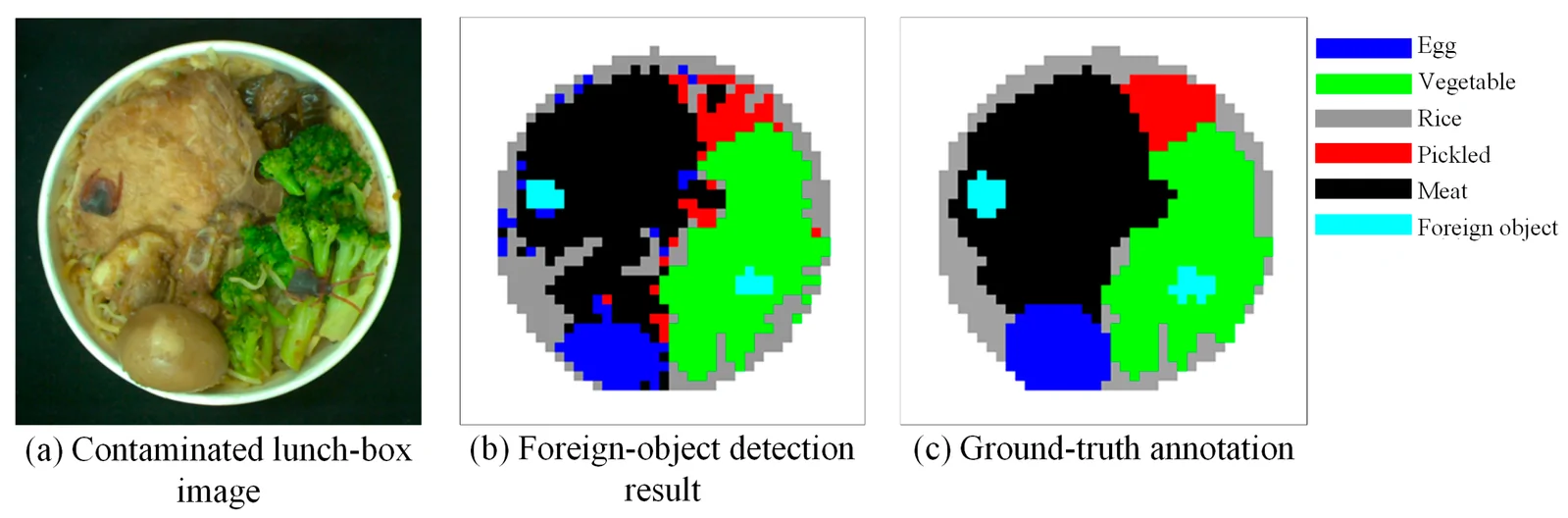
Foreign-object detection results obtained using the proposed inspection system.

As illustrated in Figure 22a, dismembered plastic cockroaches were placed in different locations inside a lunchbox to simulate contamination. After food classification, regions identified as foreign objects were labeled in sky blue. Detection performance was evaluated by comparing the detected foreign-object region with the manually annotated ground truth (Figure 22c). A foreign object was considered successfully detected when more than 50% of its manually annotated area was correctly identified. This threshold was adopted as a practical engineering criterion to determine whether a lunch box should be classified as contaminated. Since the objective of the proposed system is lunch-box-level defect detection rather than precise pixel-level segmentation, identifying the majority of the foreign-object region was considered sufficient for reliable inspection while reducing the influence of minor boundary discrepancies and isolated misclassified regions.

In this study, foreign-object contamination was simulated using a plastic cockroach model as a proof-of-concept case. A foreign object was considered successfully detected when more than 50% of its manually annotated area was classified as foreign-object superpixels. This threshold was adopted as an empirical operational criterion rather than a regulatory or universally established food-safety threshold. Because the fixed-grid representation may introduce boundary-level disagreement between the detected and manually annotated regions, the criterion was used to determine whether a sufficient portion of the foreign object was identified to trigger an image-level defect decision. Validation of this threshold for practical food-safety applications requires a larger dataset containing foreign objects with different materials, sizes, colors, and degrees of occlusion.

## 4. Experiments, Results, and Discussion

To evaluate the effectiveness of the proposed vision-based lunch-box inspection system, comprehensive experiments were conducted to assess its performance in food classification, portion defect detection, and foreign-object detection. The evaluation consisted of parameter optimization, large-scale performance testing, comparative analysis, and feasibility assessment under practical operating conditions.

### 4.1. Implementation of the Inspection System

The proposed inspection system was implemented on a personal computer equipped with an AMD Ryzen™ 7 1700 CPU (3.7 GHz), 16 GB RAM, an NVIDIA GeForce GTX 1070 GPU, and Windows 10. The image acquisition system consisted of a Basler 1.3-megapixel color CCD camera, a 10 mm lens, and an overhead ring-light illumination system, providing stable imaging conditions for automated lunch-box inspection.

**Figure 23:**
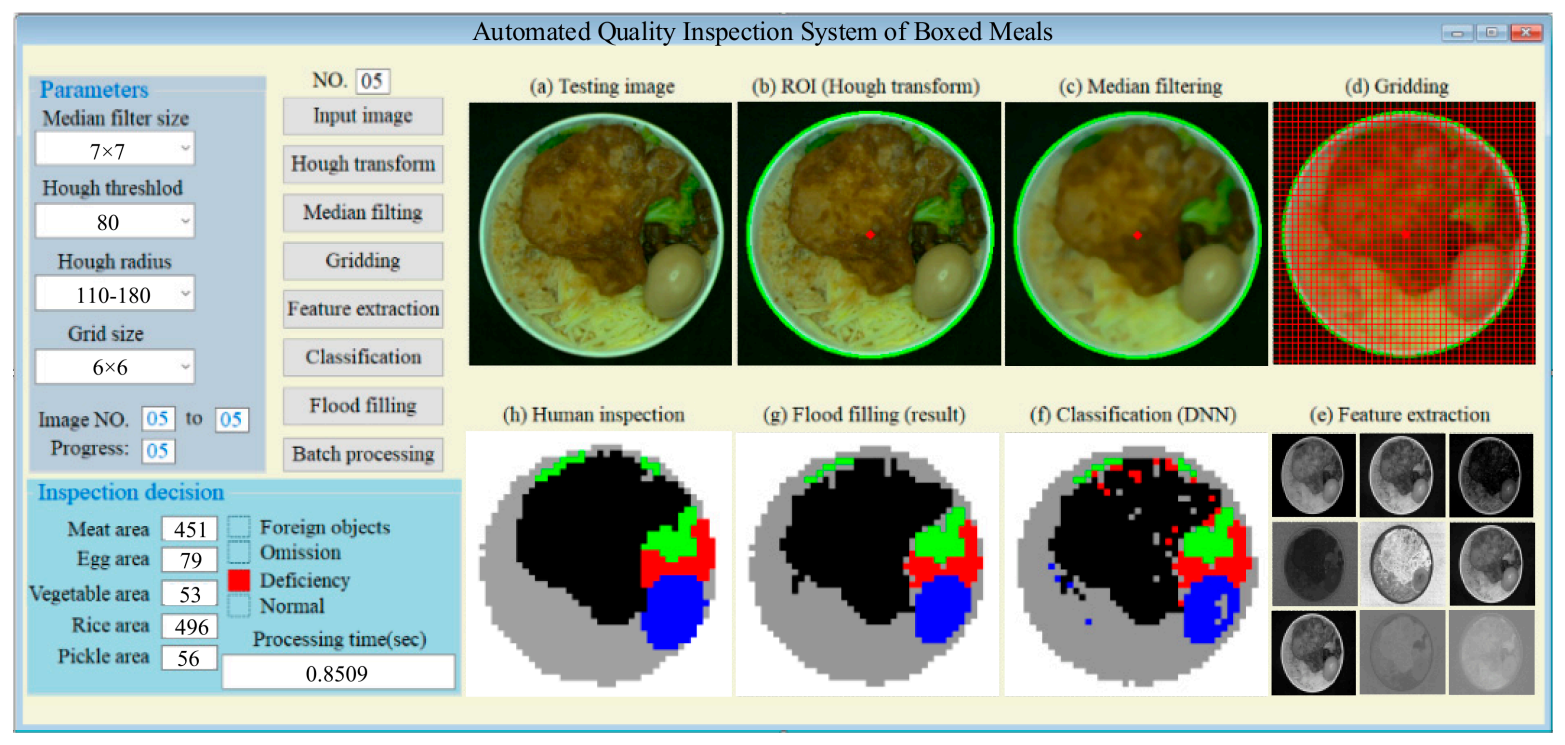
Graphical user interface of the proposed lunch-box inspection system. In the classification and inspection-result images, black, blue, green, red, and gray represent meat, egg, vegetables, pickled side dish, and rice, respectively. The colored indicators in the inspection-decision panel denote the corresponding inspection status.

The inspection software was developed in Python 3.6 using the TensorFlow 1.0, Keras 1.2, and PyQt5 libraries. Input images were cropped to a square format and resized to 256 × 256 pixels before processing. As shown in Figure 23, the system performs ROI extraction using the Hough Transform, applies median filtering to suppress reflective noise, partitions the ROI into superpixels, extracts color features, and classifies each superpixel using the proposed DNN model. Flood-filling post-processing is subsequently applied to refine the classification results. Finally, the system evaluates food portions, detects foreign objects, and determines whether the lunch box is classified as normal, missing food (omission), insufficient portion (deficiency), or foreign-object contamination. The GUI also displays the estimated area of each food category and the total processing time, providing an integrated platform for automated lunch-box quality inspection. 

### 4.2. Performance Evaluation Metrics

The performance of the proposed lunch-box inspection system was evaluated by comparing the detection results with manually annotated ground-truth images. Two levels of evaluation were adopted: superpixel-level evaluation for food classification and foreign-object detection, and image-level evaluation for lunch-box defect detection. The defined metrics were subsequently used for parameter optimization, method comparison, and overall performance assessment.

#### 4.2.1. Superpixel-Level Performance Evaluation

Foreign-object detection and food classification were evaluated at the superpixel level, where each superpixel was assigned the class label corresponding to the dominant pixel category in the manually annotated ground-truth image. Four performance metrics were employed: (1) foreign-object detection rate, (2) food false detection rate, (3) food misclassification rate, and (4) superpixel classification accuracy, as defined in Equations (3)–(6).

1. Foreign-object detection rate

(3) $$P\text{\_}1-\beta \;(\%)=\frac{\mathrm{Number}\;(\mathrm{area})\;\mathrm{of}\;\mathrm{correctly}\;\mathrm{detected}\;\mathrm{foreign}-\mathrm{object}\;\mathrm{superpixels}}{\mathrm{Number}\;(\mathrm{area})\;\mathrm{of}\;\mathrm{actual}\;\mathrm{foreign}-\mathrm{object}\;\mathrm{superpixels}}\times 100\%$$

2. Food false detection rate

(4) $$P\text{\_}\alpha \;(\%)=\frac{\mathrm{Number}\;(\mathrm{area})\;\mathrm{of}\;\mathrm{food}\;\mathrm{superpixels}\;\mathrm{misclassified}\;\mathrm{as}\;\mathrm{foreign}\;\mathrm{objects}}{\mathrm{Number}\;(\mathrm{area})\;\mathrm{of}\;\mathrm{actual}\;\mathrm{food}\;\mathrm{superpixels}}\times 100\%$$

3. Food misclassification rate

(5) $$P\text{\_}\gamma \;(\%)=\frac{\mathrm{Number}\;(\mathrm{area})\;\mathrm{of}\;\mathrm{incorrectly}\;\mathrm{classified}\;\mathrm{food}\;\mathrm{superpixels}}{\mathrm{Number}\;(\mathrm{area})\;\mathrm{of}\;\mathrm{actual}\;\mathrm{food}\;\mathrm{superpixels}}\times 100\%$$

4. Superpixel classification accuracy

(6) $$\text{P\_CR}\;(\%)=\frac{\mathrm{Number}\;(\mathrm{area})\;\mathrm{of}\;\mathrm{correctly}\;\mathrm{classified}\;\mathrm{food}\;\mathrm{and}\;\mathrm{foreign}-\mathrm{object}\;\mathrm{superpixels}}{\mathrm{Total}\;\mathrm{number}\;(\mathrm{area})\;\mathrm{of}\;\mathrm{superpixels}}\times 100\%$$

#### 4.2.2. Image-Level Performance Evaluation

Overall lunch-box inspection performance was evaluated at the image level. Defective images included lunch boxes containing foreign objects, missing food items, or insufficient food portions. A foreign object was regarded as successfully detected when at least 50% of its ground-truth area was correctly identified. Food defects were determined by comparing the measured food area with the empirical food-quantity thresholds described in Section 3.8. Four image-level metrics were adopted: (1) defective image detection rate, (2) false alarm rate for normal images, (3) defective image misclassification rate, and (4) overall image classification rate, as defined in Equations (7)–(10).

1. Defective image detection rate

(7) $$1-\beta \;(\%)=\frac{\mathrm{Number}\;\mathrm{of}\;\mathrm{correctly}\;\mathrm{classified}\;\mathrm{defective}\;\mathrm{images}}{\mathrm{Number}\;\mathrm{of}\;\mathrm{actual}\;\mathrm{defective}\;\mathrm{images}}\times 100\%$$

2. Normal-image false alarm rate

(8) $$\alpha \;(\%)=\frac{\mathrm{Number}\;\mathrm{of}\;\mathrm{normal}\;\mathrm{images}\;\mathrm{classified}\;\mathrm{as}\;\mathrm{defective}}{\mathrm{Number}\;\mathrm{of}\;\mathrm{actual}\;\mathrm{normal}\;\mathrm{images}}\times 100\%$$

3. Defective image misclassification rate

(9) $$\gamma \;(\%)=\frac{\mathrm{Number}\;\mathrm{of}\;\mathrm{incorrectly}\;\mathrm{classified}\;\mathrm{defective}\;\mathrm{images}}{\mathrm{Number}\;\mathrm{of}\;\mathrm{actual}\;\mathrm{defective}\;\mathrm{images}}\times 100\%$$

4. Overall image classification rate

(10) $$\mathrm{CR}\;(\%)=\frac{\mathrm{Number}\;\mathrm{of}\;\mathrm{correctly}\;\mathrm{classified}\;\mathrm{normal}\;\mathrm{and}\;\mathrm{defective}\;\mathrm{images}}{\mathrm{Total}\;\mathrm{number}\;\mathrm{of}\;\mathrm{images}}\times 100\%$$

### 4.3. Parameter Optimization

The proposed inspection system includes several parameters that require optimization, including the minimum and maximum radii and accumulator threshold of the Hough Transform for ROI extraction, the superpixel grid size, the number of hidden layers and neurons in the DNN classifier, and the empirical decision thresholds for food-portion evaluation. The optimal parameter settings were determined using the performance metrics described in Section 4.2.

The complete dataset consisted of 667 lunch-box images, including 350 normal images and 317 defective images. The dataset was divided into training (310 images), validation (90 images), and testing (267 images) subsets, as summarized in Table 3. The training dataset was used to train the DNN classifier and determine the empirical decision thresholds, while the validation dataset was used for parameter optimization, including the selection of the superpixel size, DNN architecture, classifier parameters, and selection of empirical decision thresholds. The independent testing dataset was reserved exclusively for the final performance evaluation of the proposed inspection framework. All images were resized to 256 × 256 pixels before analysis. The defective samples included missing food items, insufficient food portions, and foreign-object contamination.

#### 4.3.1. Optimization of ROI Extraction Parameters

The circular Hough transform parameters were optimized to achieve reliable ROI extraction for lunch-box images. Because the lunch-box container radius was approximately 115 pixels in the resized 256 × 256 images, the search range was fixed at *Min_R* = 100 pixels and *Max_R* = 130 pixels. The accumulator threshold (*P_1_*) was then evaluated using values of 15, 20, 30, and 40, as summarized in Table 4.

**Figure 24:**
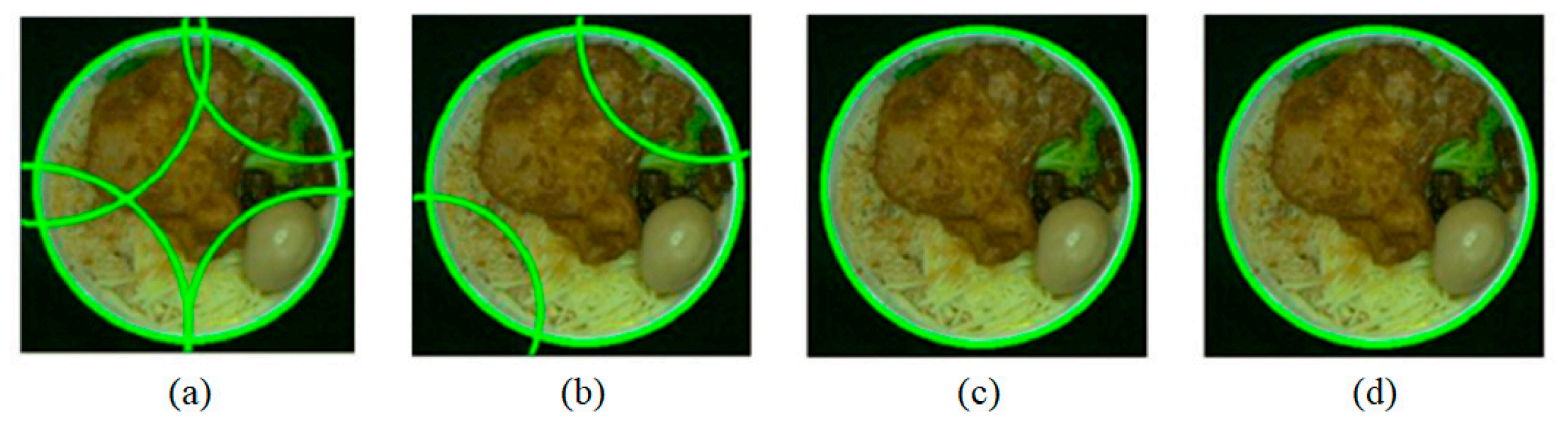
Effect of the accumulator threshold (*P*_1_) on ROI extraction using the Circular Hough Transform. (**a**) *P*_1_ = 15; (**b**) *P*_1_ = 20; (**c**) *P*_1_ = 30; (**d**) *P*_1_ = 40. Increasing *P*_1_ suppresses spurious circle detections, with *P*_1_ = 40 providing the highest mean IoU (0.9642).

To quantitatively evaluate the effect of the accumulator threshold *P*_1_ on ROI localization, the automatically detected circular lunch-box region was compared with the manually defined reference ROI using the Intersection-over-Union (IoU) metric across the training and validation images. As summarized in Table 4, the mean IoU increased substantially with *P*_1_, from 0.4067 at *P*_1_ = 15 and 0.5443 at *P*_1_ = 20 to 0.8874 at *P*_1_ = 30 and 0.9642 at *P*_1_ = 40. The relatively low IoU values at *P*_1_ = 15 and *P*_1_ = 20 were primarily associated with multiple spurious circle detections, as illustrated in Figure 24a,b. Increasing the threshold to *P*_1_ = 30 substantially suppressed these false detections and improved localization accuracy; however, occasional extraneous circular responses remained, as shown in Figure 24c. At *P*_1_ = 40, the detected boundary closely matched the lunch-box boundary, resulting in the highest mean IoU of 0.9642. Therefore, *P*_1_ = 40 was selected for ROI extraction in all subsequent experiments based on both the quantitative IoU evaluation and the representative visual results shown in Figure 24. 

#### 4.3.2. Superpixel Size Selection

The superpixel size affects both classification accuracy and computational efficiency. Small superpixels preserve fine image details but increase computational complexity, whereas large superpixels reduce processing time at the expense of boundary accuracy and local feature representation. To determine the optimal configuration, superpixel sizes ranging from 5 × 5 to 9 × 9 pixels were evaluated.

**Figure 25:**
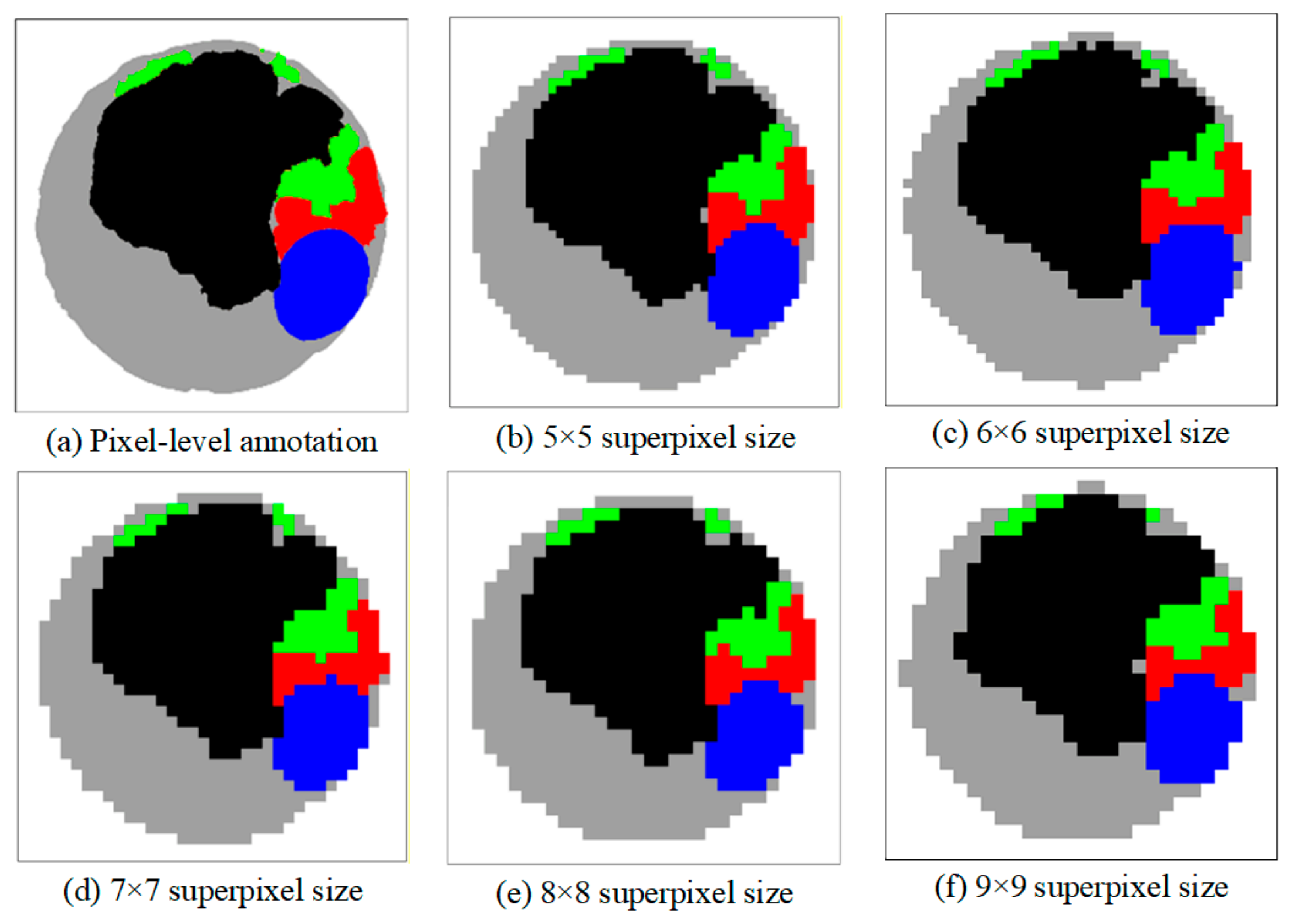
Manual food-region annotations using different superpixel sizes. Black, blue, green, red, and gray represent meat, egg, vegetables, pickled side dish, and rice, respectively.

**Figure 26:**
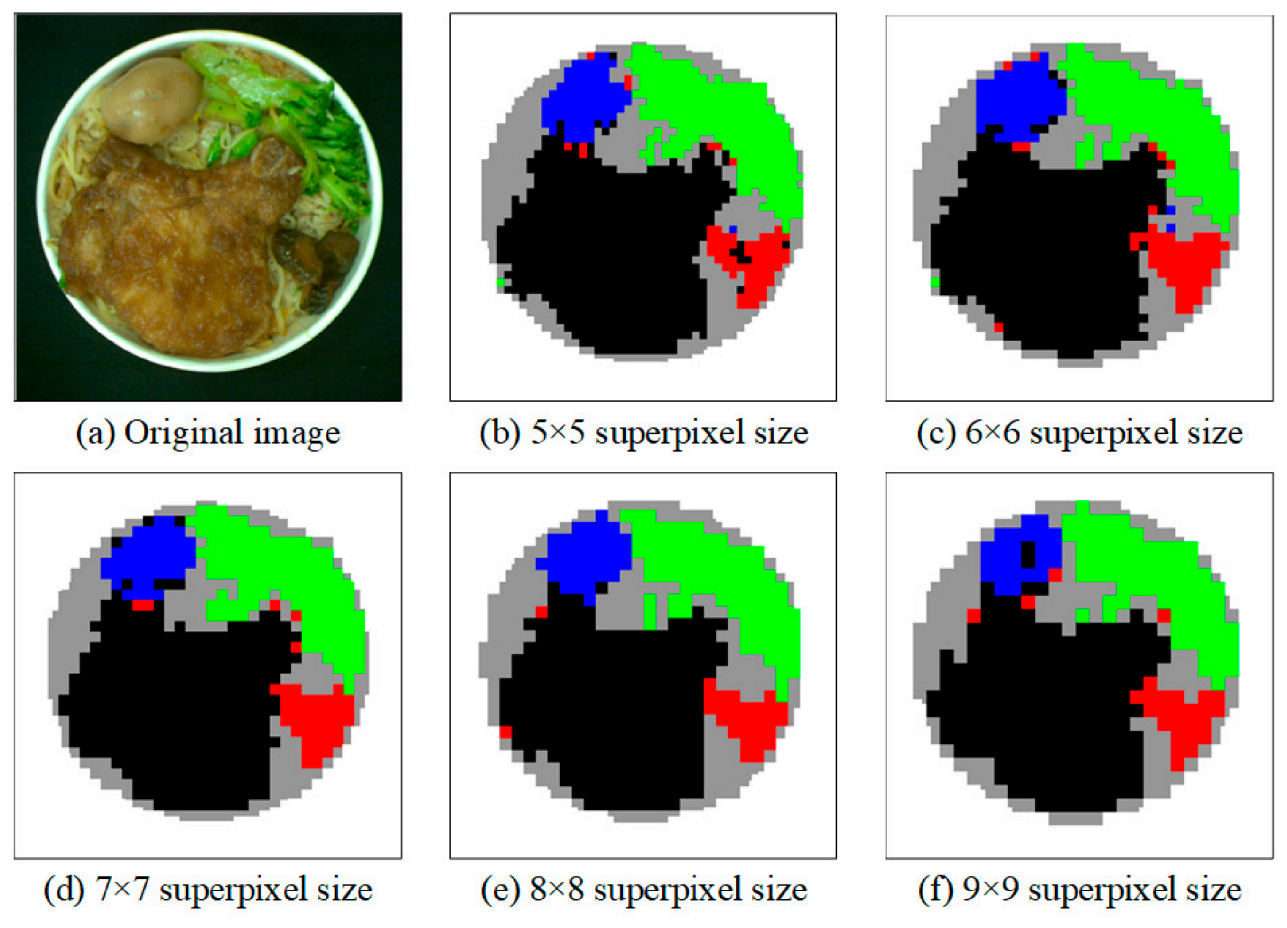
Food classification results using different superpixel sizes. Black, blue, green, red, and gray represent meat, egg, vegetables, pickled side dish, and rice, respectively.

Figure 25 compares the corresponding manual annotations. As the superpixel size increased, food boundaries became progressively coarser, resulting in reduced boundary accuracy. Figure 26 presents the corresponding food classification results. The 6 × 6 superpixel size produced the best visual agreement with the ground truth, whereas larger superpixel sizes (8 × 8 and 9 × 9) caused noticeable boundary distortion and loss of fine details.

The quantitative results are summarized in Table 5. Among the evaluated configurations, the 6 × 6 superpixel size achieved the highest superpixel classification accuracy (89.17%), the highest foreign-object detection rate (81.62%), and the lowest food misclassification rate (10.83%). Although the 9 × 9 configuration yielded the lowest food false-positive rate (0.10%), its overall classification accuracy decreased because of reduced spatial resolution. Therefore, a 6 × 6 superpixel size was selected for all subsequent experiments.

The selection of the superpixel size was based on a practical trade-off between classification performance and computational efficiency. Smaller superpixels preserved more detailed boundary information but increased the computational burden owing to the larger number of regions requiring feature extraction and classification. In contrast, larger superpixels reduced computational complexity but tended to smooth object boundaries and increase classification errors. Therefore, the 6 × 6 superpixel size was selected because it provided the most favorable overall balance between recognition accuracy and processing efficiency among the evaluated configurations.

#### 4.3.3. DNN Architecture Optimization

To determine the optimal DNN architecture, the number of hidden layers (*L*) and the number of neurons per hidden layer (*N*) were systematically evaluated, while the remaining training parameters were kept fixed as summarized in Table 6. Each input sample consisted of an 18-dimensional feature vector comprising the mean and standard deviation of the RGB, HSV, and CIE Lab* components extracted from a fixed-grid region. The output layer contained six neurons corresponding to meat, egg, vegetable, pickled side dish, rice, and foreign object. ReLU activation was used in the hidden layers, Softmax was applied to the output layer, categorical cross-entropy was adopted as the loss function, and the Adam optimizer was used for model training.

The DNN training and reproducibility settings were specified as follows. The selected DNN consisted of an 18-dimensional input layer, four fully connected hidden layers with 40 neurons per layer, and a six-neuron output layer, resulting in 5926 trainable parameters, including bias terms. The network was trained using the Adam optimizer with an initial learning rate of 0.001 and categorical cross-entropy loss. A maximum of 100 epochs and a batch size of 200 were used. Early stopping was not applied because all candidate architectures were trained using the same fixed number of epochs to ensure a consistent comparison during architecture optimization. The training and validation loss curves were monitored throughout training to verify convergence. For reproducibility, the random seed was fixed at 42 for network initialization and data shuffling. No class weighting or resampling was applied, and the original class distribution was retained during training.

**Figure 27:**
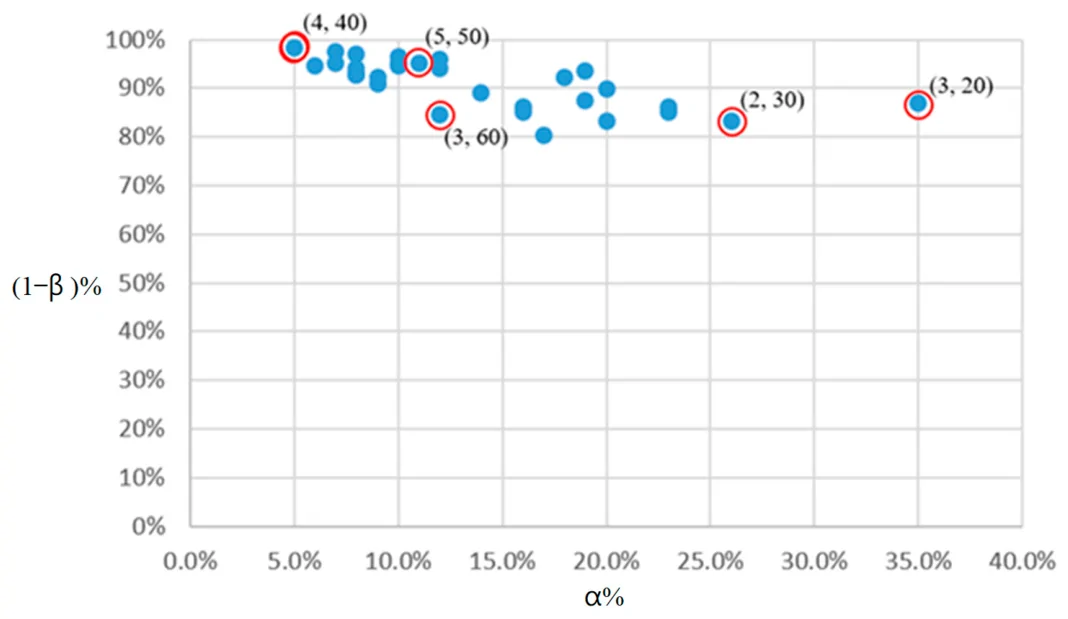
ROC analysis of first-stage DNN architecture optimization based on defective-image detection rate (1−β)% and false alarm rate α%.

**Figure 28:**
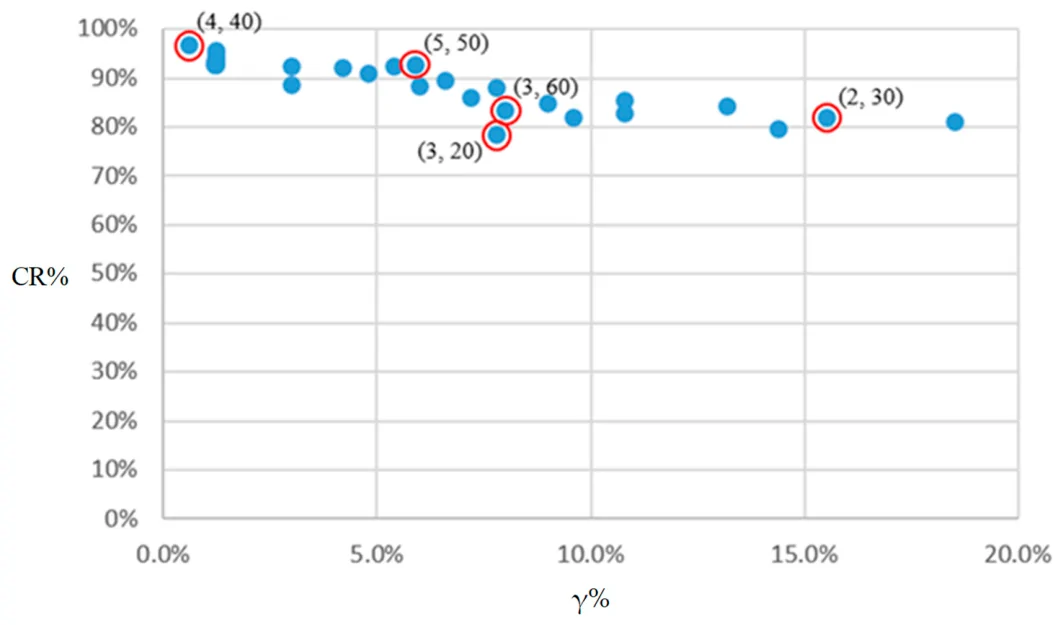
ROC analysis of first-stage DNN architecture optimization based on overall image classification rate CR% and defective-image misclassification rate γ%.

In the first-stage optimization, *L* was varied from 1 to 6, while *N* was varied from 20 to 60 in increments of 10, resulting in 30 candidate architectures (Table 7). Their performance was evaluated using the defective-image detection rate (1−β)%, normal-image false alarm rate (α%), defective-image misclassification rate (γ%), and overall image classification rate (CR%), as summarized in Table 8 and Figure 27 and Figure 28. Among the evaluated configurations, (*L*, *N*) = (4, 40) provided the most favorable empirical trade-off among the considered performance metrics. 

A second-stage optimization was subsequently conducted by fixing *L* = 4 and varying *N* from 30 to 50 in increments of 5. As shown in Table 9, *N* = 40 again provided the most favorable overall performance, with a defective-image detection rate of 98.20%, a false alarm rate of 5.00%, a defective-image misclassification rate of 0.60%, and an overall classification rate of 97.00%. The final DNN therefore consisted of an 18-neuron input layer, four fully connected hidden layers containing 40 neurons each, and a six-neuron Softmax output layer. Including the bias terms, the selected architecture contained 5926 trainable parameters and was adopted in all subsequent experiments.

#### 4.3.4. Selection of Food-Omission and Insufficient-Portion Thresholds

Following food classification, an empirical threshold determination approach was employed to determine whether each food item was missing or insufficient. The mean (μ) and standard deviation (σ) of the food area, measured in superpixels, were calculated from the training dataset to establish category-specific decision thresholds. The training samples used for threshold estimation are summarized in Table 10. Because braised eggs are discrete objects that are frequently occluded by the pork chop, only egg omission was considered. Similarly, due to the large variation in the size and shape of pickled cucumber, only pickle omission was evaluated.

**Figure 29:**
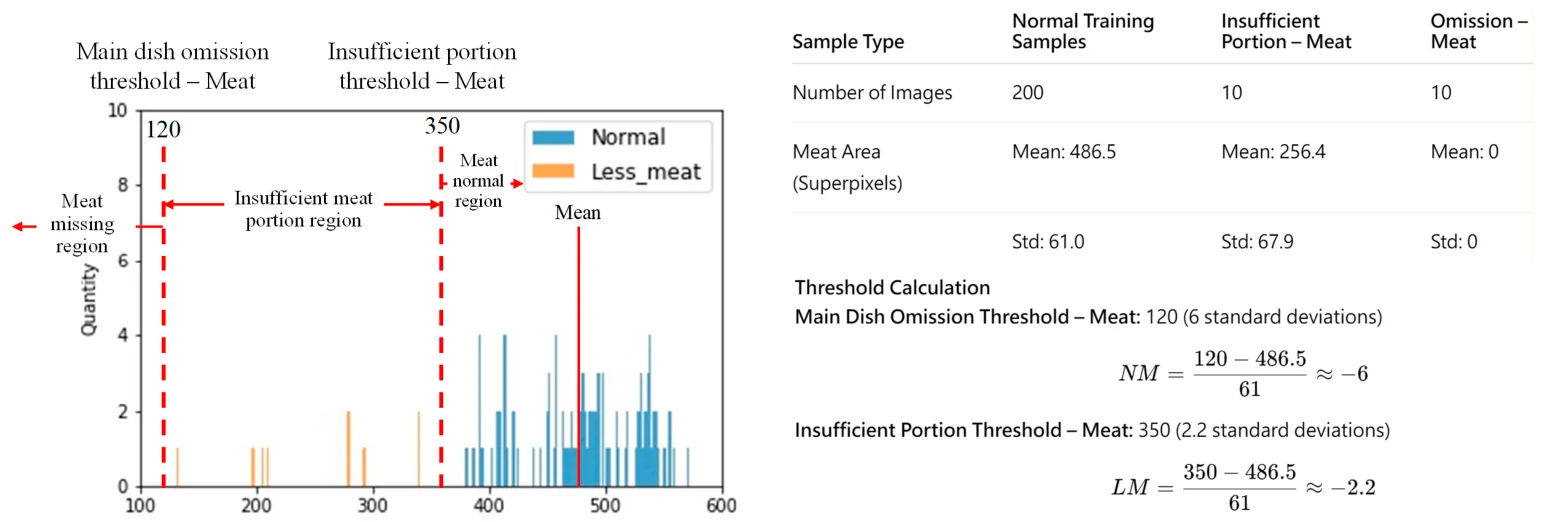
Example of empirical food-quantity threshold determination for meat omission and insufficient-portion detection. The red dashed vertical lines indicate the empirically determined thresholds for meat omission (120 superpixels) and insufficient portion (350 superpixels), while the horizontal arrows indicate the corresponding meat-missing, insufficient-portion, and normal-portion regions. The solid red vertical line indicates the mean meat area of the normal training samples.

Thresholds were determined by analyzing the area distributions of normal and defective samples. Figure 29 illustrates the threshold selection procedure for meat omission and insufficient-portion detection. Three common fixed-threshold groups were also evaluated to assess whether a uniform, normalized threshold could be applied across food categories. As shown in Table 11, the category-specific strategy provided better overall performance, indicating that differences in the area distributions of individual food categories should be considered. However, the resulting thresholds are empirical parameters calibrated for the investigated lunch-box configuration and should be recalibrated when the menu composition or nominal portion specifications change.

The proposed method consistently outperformed the fixed-threshold strategies, achieving a defective-image detection rate of 98.20%, an overall classification accuracy of 97.00%, and a defective-image misclassification rate of only 0.60%. Although its false alarm rate (5.00%) was slightly higher than those of Parameter Groups 1 and 2, it substantially improved defective-image detection. It should be noted that the proposed threshold coefficients were determined empirically from the observed training-data distributions rather than derived from a parametric statistical model. The mean and standard deviation were used only to normalize the thresholds, facilitating comparison among different food categories.

### 4.4. Comparison of Supervised Classifiers

To evaluate the effectiveness of the proposed DNN, its performance was compared with two widely used supervised classifiers: Support Vector Machine (SVM) and Random Forest (RF). The comparison was conducted in two stages. First, the training dataset was used to determine the optimal parameters of the SVM and RF classifiers. Second, the optimized classifiers were evaluated on the complete testing dataset and compared with the proposed DNN.

**Figure 30:**
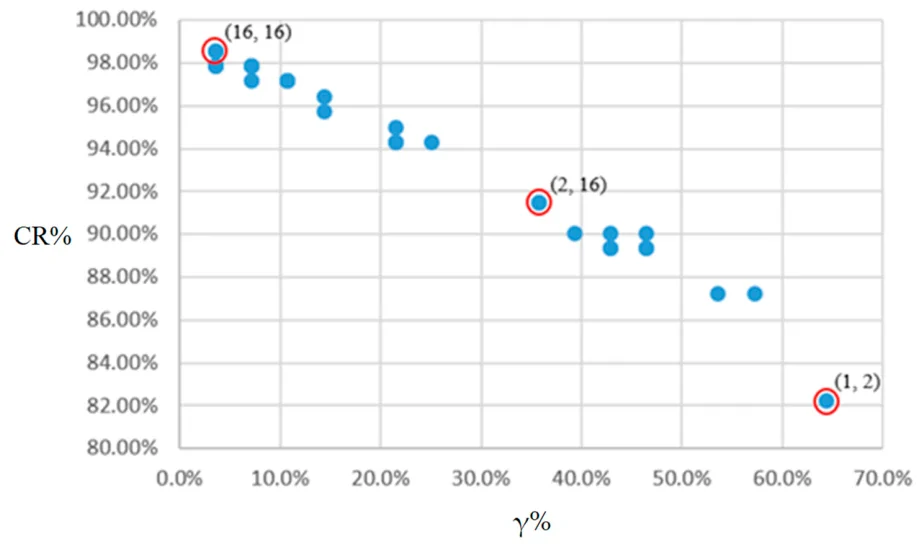
ROC-based optimization of SVM parameters using the training dataset.

**Figure 31:**
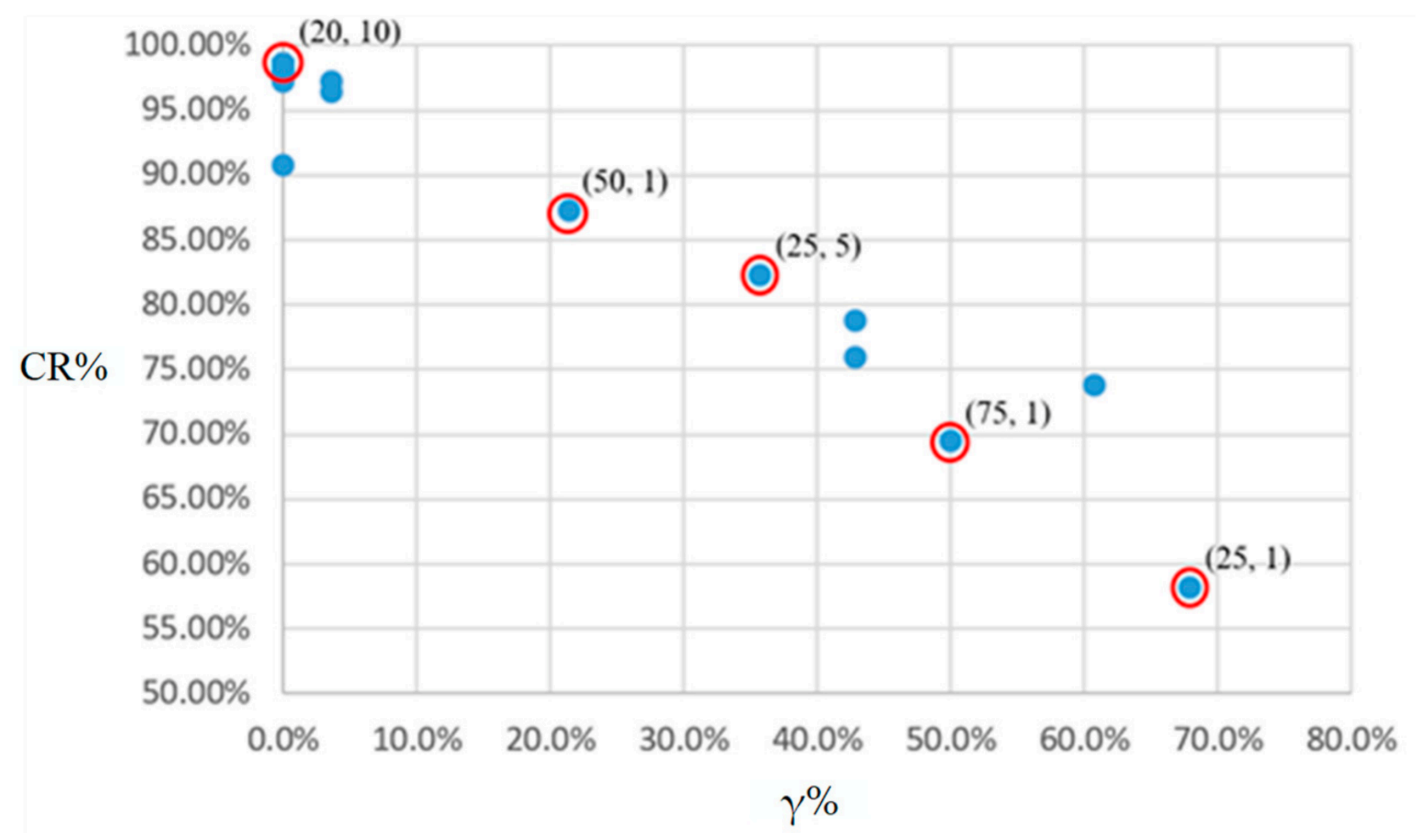
ROC-based optimization of Random Forest parameters using the training dataset.

For SVM with the Radial Basis Function (RBF) kernel, the penalty parameter (*C*) and gamma parameter (*gamma*) were optimized using ROC analysis. As shown in Figure 30, the optimal configuration was *C* = 16 and *gamma* = 16. For RF, the number of trees (*n*) and maximum tree depth (*d*) were optimized, and the best performance was obtained with *n* = 20 and *d* = 10, as shown in Figure 31. 

To reduce the influence of a single data partition and assess classifier stability, five-fold cross-validation was performed for SVM, Random Forest, and the proposed DNN using the same evaluation protocol. Table 12 reports the performance as the mean ± standard deviation across the five folds. The proposed DNN achieved the highest superpixel classification accuracy (P_CR % = 89.46 ± 0.88%), defective-image detection rate ((1−β)% = 97.76 ± 1.21%), and overall image classification rate (CR % = 96.45 ± 1.08%), compared with CR % values of 90.25 ± 1.72% for SVM and 86.82 ± 2.26% for Random Forest. The DNN also yielded the lowest defective-image misclassification rate (γ % = 0.82 ± 0.24%) and a short processing time of 0.084 ± 0.006 s/image. The relatively small variations across the five folds indicate that the proposed DNN maintained consistent performance across different data partitions, supporting its selection as the classifier for the proposed inspection framework. 

**Figure 32:**
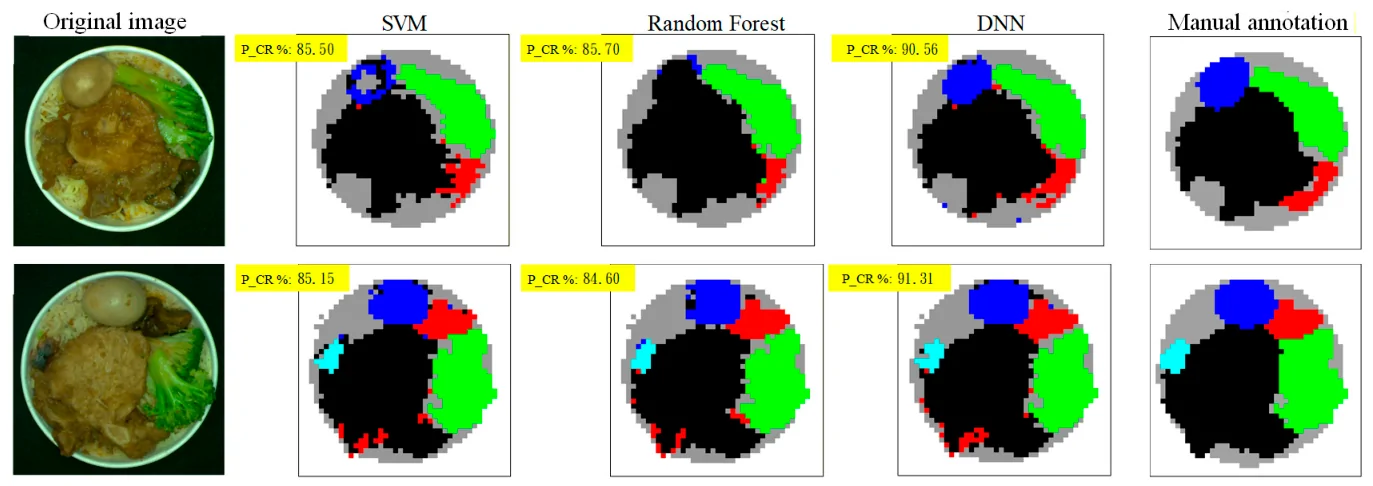
Representative food classification results obtained using SVM, Random Forest, and the proposed DNN, compared with the manually annotated ground truth. Black, blue, green, red, gray, and cyan represent meat, egg, vegetables, pickled side dish, rice, and foreign object, respectively. The yellow boxes indicate the superpixel classification accuracy (P_CR %) obtained by each classifier for the corresponding image.

Representative classification results are presented in Figure 32. Compared with SVM and RF, the proposed DNN generated smoother food boundaries, fewer isolated misclassified regions, and better agreement with the manually annotated ground truth. These qualitative observations are consistent with the quantitative results, demonstrating that the proposed DNN provides the best balance between detection accuracy and computational efficiency for lunch-box inspection. 

### 4.5. Confusion Matrix Analysis

**Figure 33:**
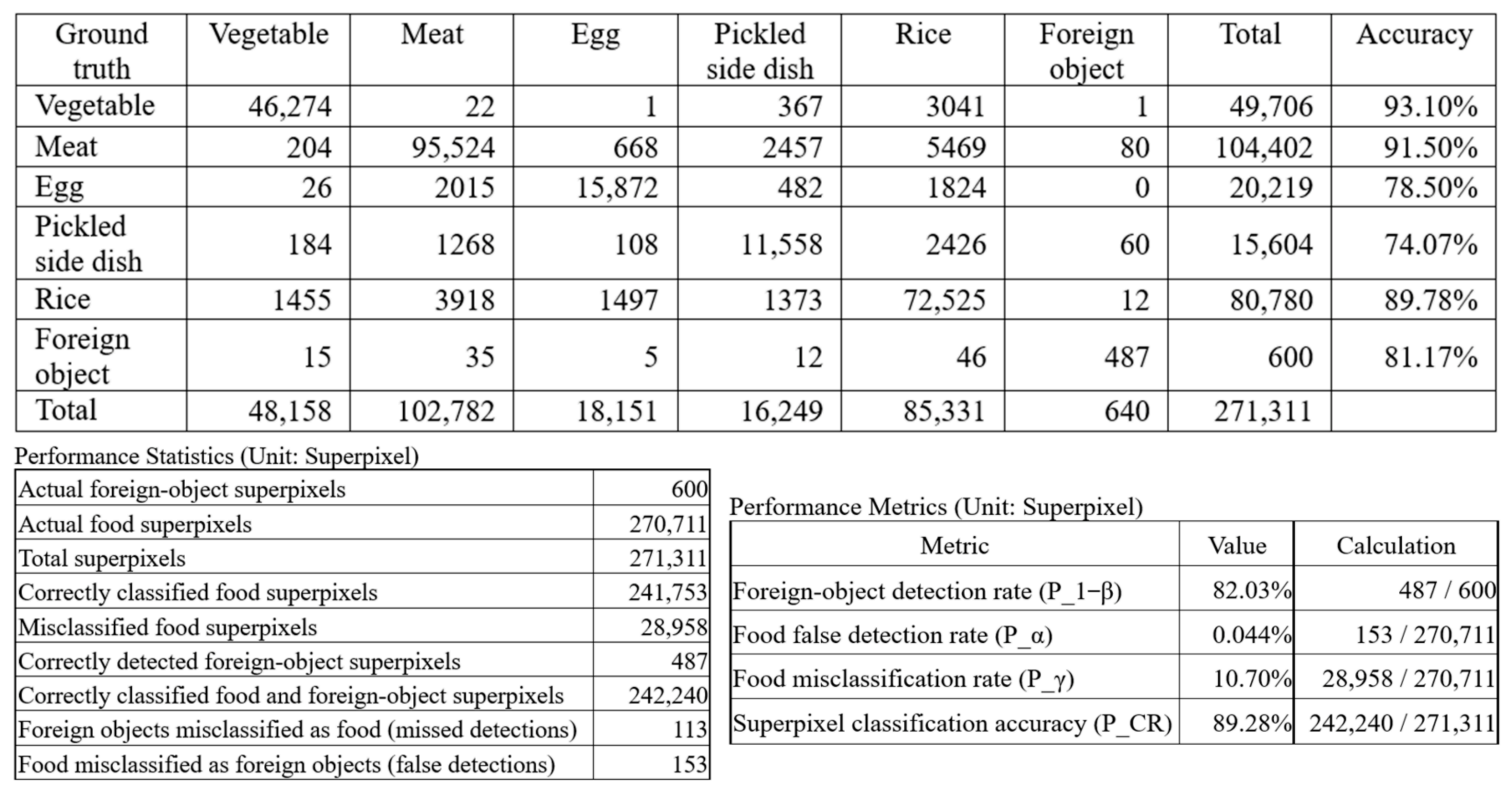
Superpixel-level confusion matrix and derived performance metrics for food classification and foreign-object detection.

To provide a comprehensive evaluation of the proposed lunch-box inspection system, confusion matrices were constructed at both the superpixel and image levels. The diagonal elements represent correctly classified samples, whereas the off-diagonal elements indicate misclassifications. Figure 33 presents the superpixel-level confusion matrix for the six food categories (meat, egg, vegetables, pickled side dish, rice, and foreign object). The corresponding performance statistics, including the foreign-object detection rate, food false positive rate, food misclassification rate, and overall superpixel classification accuracy, were calculated directly from the confusion matrix.

To further investigate whether class-dependent classification errors were associated with a particular data partition, the class-wise superpixel classification performance of the proposed DNN was examined separately across the five validation folds, as shown in Table 13. The results revealed a consistent performance pattern across the five folds. Vegetable and meat exhibited relatively high and stable classification rates, with mean values of 93.22 ± 0.60% and 91.52 ± 0.61%, respectively, while rice achieved 89.84 ± 0.53%. In contrast, egg and pickled side dish consistently exhibited lower classification performance, with mean values of 78.96 ± 0.87% and 74.78 ± 0.94%, respectively. Foreign-object superpixels achieved an intermediate mean classification rate of 82.12 ± 0.79%. The relatively small standard deviations for all six classes (0.53–0.94 percentage points) indicate that these class-dependent performance patterns were consistently observed across the five data partitions rather than being caused by a particular validation fold. The lower performance for egg and pickled side dish is consistent with the independent test-set confusion analysis in Figure 33 and can primarily be attributed to their relatively small regions, partial occlusion, boundary ambiguity, and color similarity to adjacent food categories. These results indicate that the observed class-dependent errors reflect recurring limitations of the fixed-grid color-based representation rather than instability associated with a specific data split.

**Figure 34:**
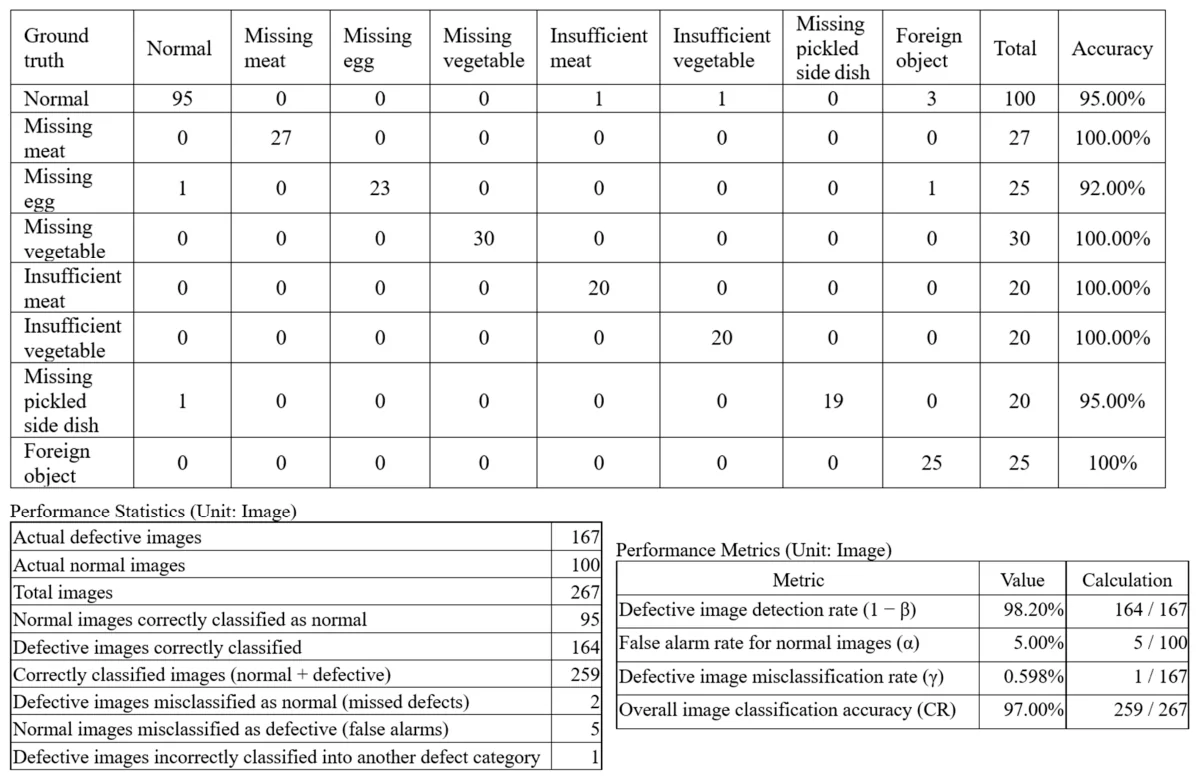
Image-level confusion matrix and derived performance metrics for lunch-box defect detection.

Figure 34 presents the image-level confusion matrix for normal lunch boxes and seven defect categories, including missing food items, insufficient food portions, and foreign-object contamination. Based on the image-level confusion matrix, the defect detection rate, false positive rate, defect misclassification rate, and overall image classification rate were computed to evaluate the practical performance of the proposed inspection system.

Figure 34 further reveals the relationship between food-region classification errors and the final image-level defect decisions. Although egg exhibited relatively lower superpixel-level classification accuracy because of its small visible area, partial occlusion by the pork chop, and color similarity with adjacent food regions, 23 of the 25 missing-egg test images were correctly identified, corresponding to an image-level accuracy of 92.0%. Similarly, 19 of the 20 missing-pickled-side-dish images were correctly classified (95.0%). In comparison, all test images representing missing meat, missing vegetable, insufficient meat, insufficient vegetable, and foreign-object defects were correctly identified. These results indicate that localized superpixel misclassification does not necessarily lead directly to an incorrect image-level defect decision because the final decision is based on the aggregated food region and its corresponding category-specific threshold. Nevertheless, smaller and partially occluded food categories remain more susceptible to classification errors, particularly near boundaries between adjacent food regions.

### 4.6. Sensitivity Experiments on Performance Stability Under Evaluated Laboratory and Prototype Conditions

To evaluate the feasibility and practical applicability of the proposed lunch-box inspection system, a series of sensitivity analyses were conducted under different operating conditions. The experiments examined the effects of variations in two representative TRA lunch-box products with different container geometries and food compositions, imaging illumination, and conveyor-based image acquisition on inspection performance. For each experimental condition, the proposed system was quantitatively evaluated using the performance metrics described in Section 4.2. These results demonstrate the adaptability of the proposed framework under the evaluated controlled lunch-box configuration.

#### 4.6.1. Adaptability and Feasibility of the Proposed Framework Under the Tested Lunch-Box Configuration

**Figure 35:**
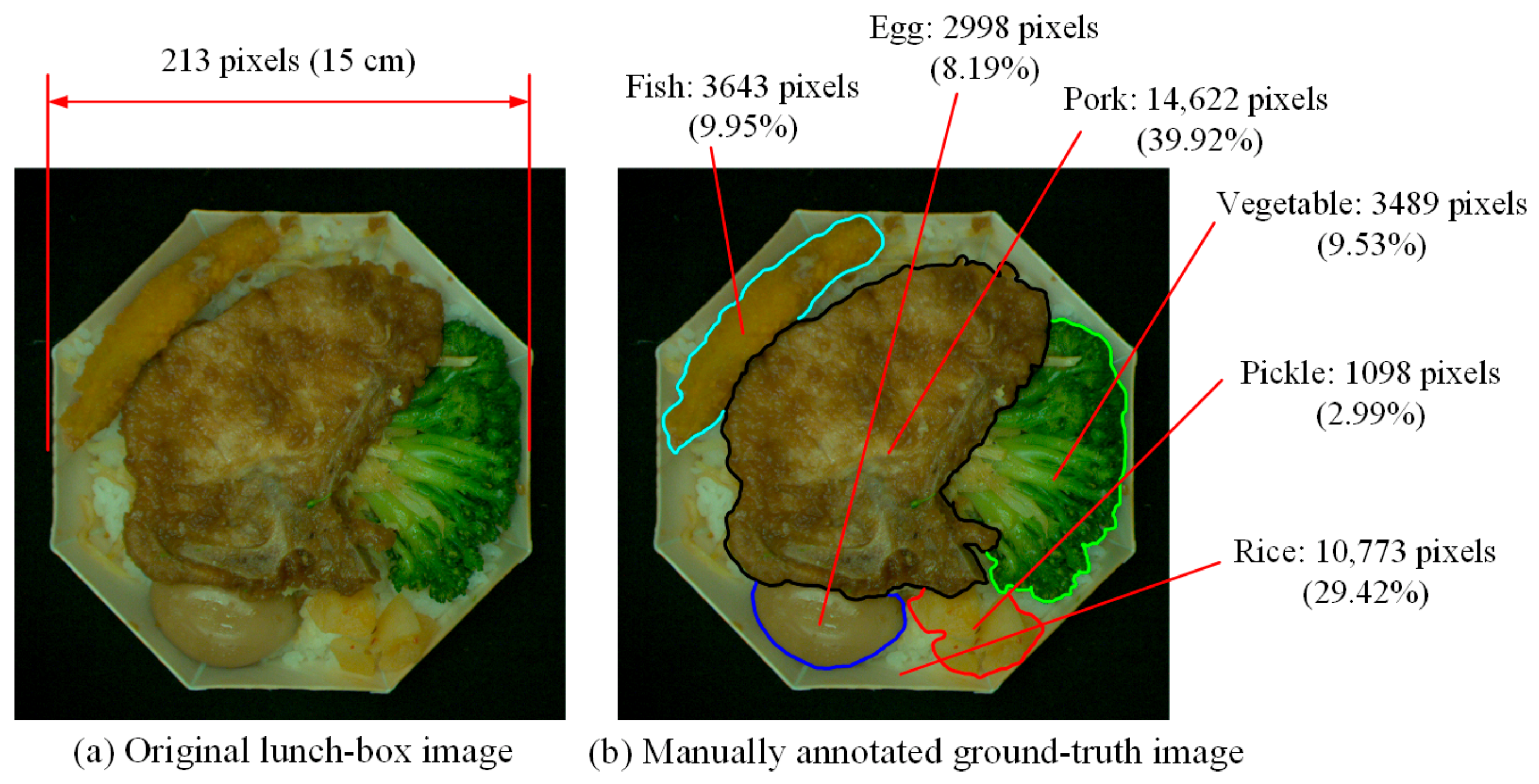
Food composition and manually annotated ground truth of the TRA octagonal pork chop lunch box.

To evaluate the adaptability and feasibility of the proposed inspection system, experiments were conducted using the TRA octagonal pork chop lunch box, which differs from the circular lunch box in both container geometry and food composition. As shown in Figure 35, the octagonal lunch box contains six food categories: pork chop, fried fish, braised egg, vegetables, pickled vegetables, and rice.

**Figure 36:**
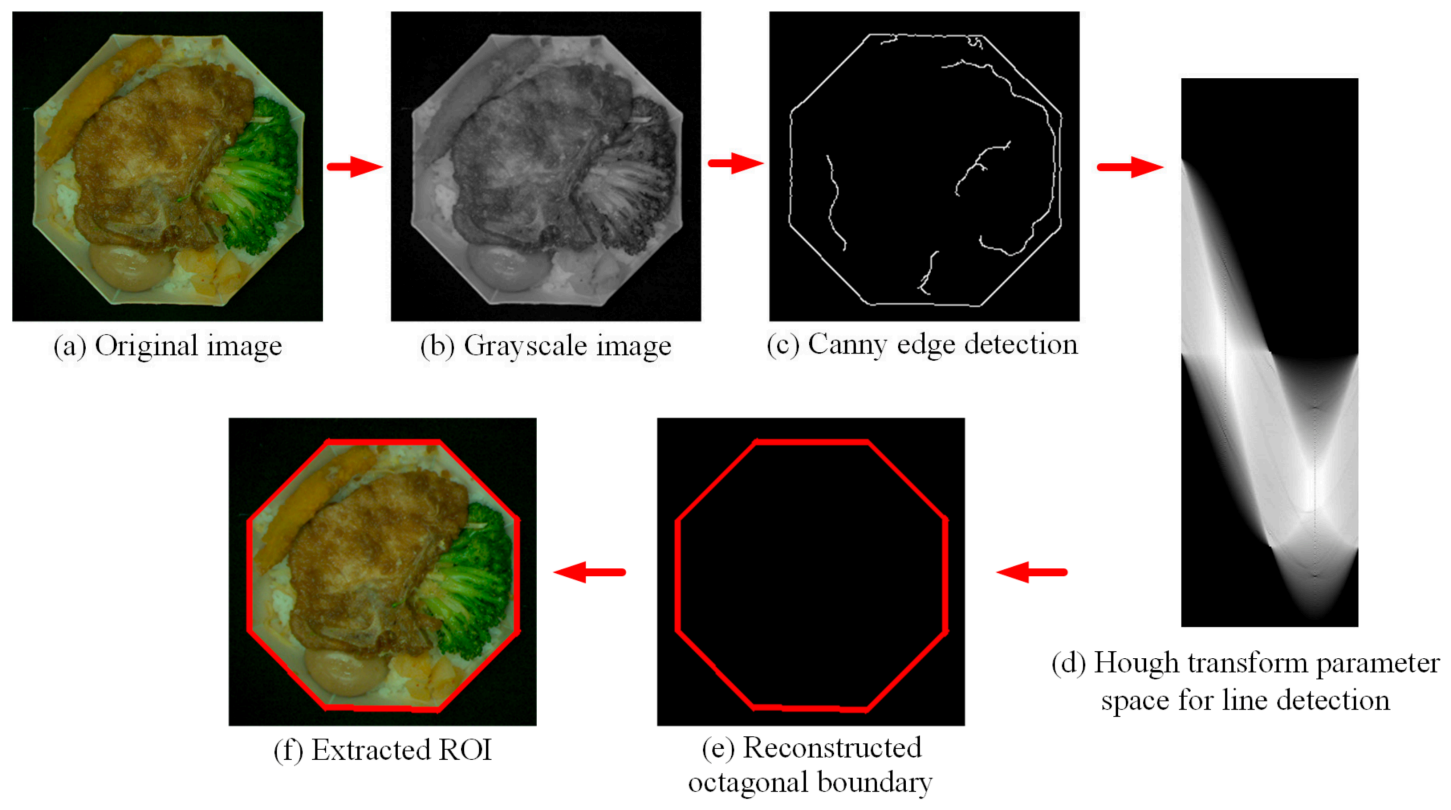
ROI extraction procedure for the TRA octagonal lunch box using the Hough transform. The red arrows indicate the sequential processing flow, while the red lines in (**e**) and (**f**) indicate the reconstructed octagonal boundary and the detected ROI boundary, respectively.

**Figure 37:**
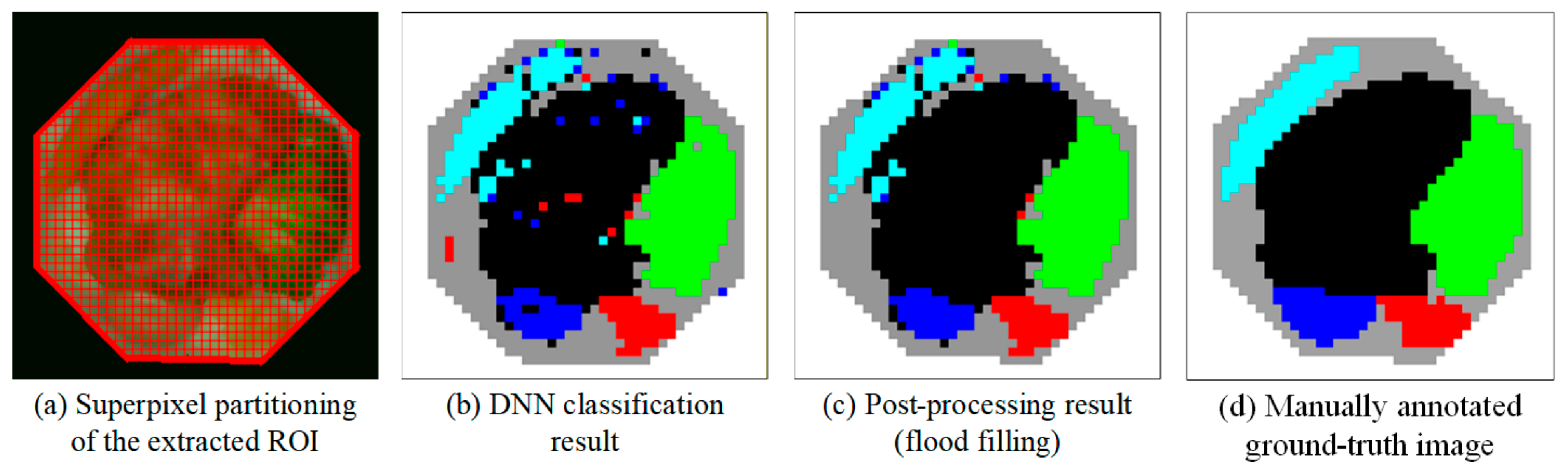
Food classification procedure for the TRA octagonal pork chop lunch box. The red lines in (**a**) indicate the superpixel boundaries. In (**b**)–(**d**), black, blue, green, red, gray, and cyan represent meat, egg, vegetables, pickled side dish, rice, and fish, respectively.

The ROI was extracted using the Hough transform for line detection. As illustrated in Figure 36, the input image was converted to grayscale, followed by Canny edge detection and Hough line detection to reconstruct the octagonal boundary. The extracted ROI was then partitioned into 6 × 6 superpixels, and color features in the RGB, HSV, and CIEL*a*b* color spaces were classified using the trained DNN. Finally, flood filling was applied to refine the classification results (Figure 37).

The proposed method successfully extracted the octagonal ROI and accurately classified the six food categories after retraining the DNN for the corresponding menu. The classification results closely matched the manually annotated ground truth, demonstrating that the proposed framework can be readily adapted to lunch boxes with different container shapes and food compositions. The corresponding confusion matrix is presented in Table 14, where high classification accuracies were achieved for the major food categories. The present experiment provides feasibility evidence for the tested lunch-box geometry rather than establishing generalization to broader meal types or industrial production environments.

The purpose of this experiment was to evaluate the adaptability of the proposed framework to a lunch box with a different container geometry and food composition. Therefore, the evaluation focused on superpixel-level food classification performance. Since a complete image-level defect dataset containing omission, insufficient-portion, and foreign-object cases was not established for the octagonal lunch box, image-level defect detection metrics were not evaluated in this experiment.

#### 4.6.2. Effect of Illumination Variations on Detection Performance

**Figure 38:**
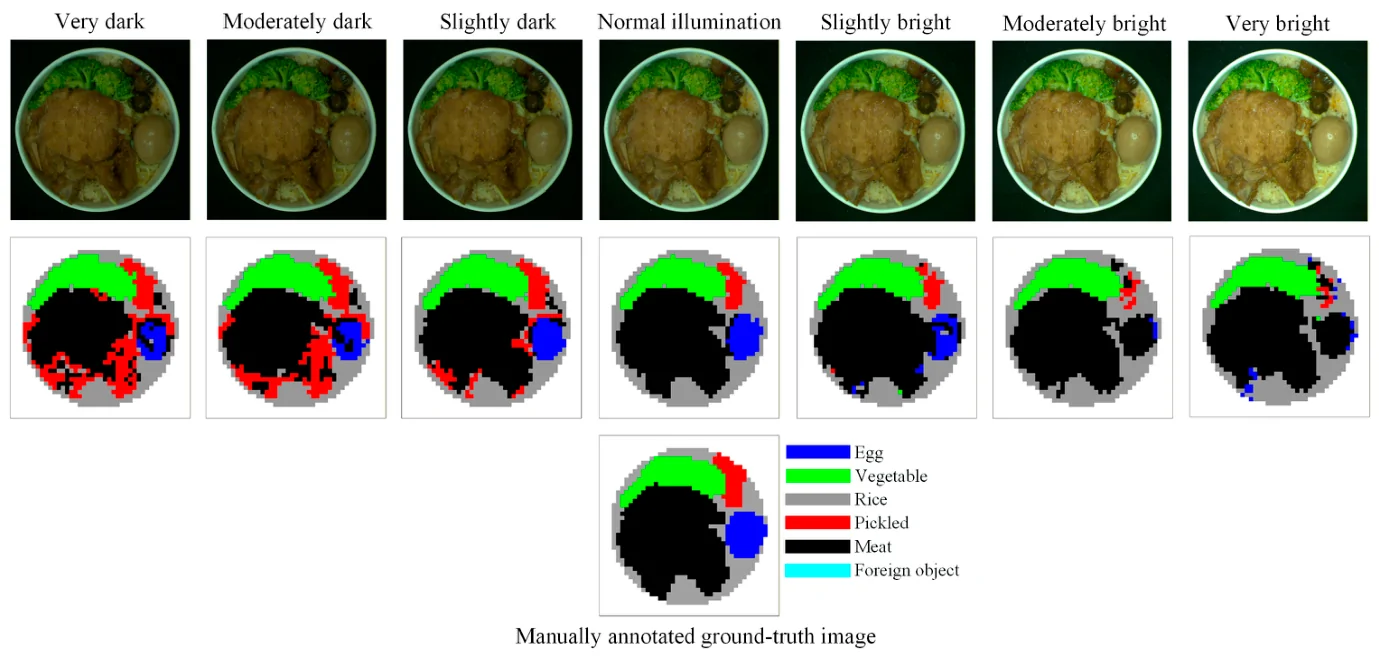
Food classification results under different illumination conditions.

To evaluate the adaptability and feasibility of the proposed inspection system under different lighting conditions, the illumination intensity of the ring LED light was adjusted using a digital LED light controller (SAKAI SX10220DG-24-4CH). Seven illumination levels, ranging from Very Dark to Very Bright, were evaluated using the TRA circular lunch box. The corresponding illumination settings, image intensity statistics, and classification accuracy are summarized in Table 15, while representative classification results are shown in Figure 38. The normal illumination level serves as the reference condition for comparison with the darker and brighter illumination levels.

In this study, the normal illumination condition refers to the standard image acquisition setting used for collecting the training, validation, and baseline testing datasets. Under this condition, the overhead ring-light illumination was operated at the nominal controller setting (LED controller setting = 20), providing uniform illumination without noticeable underexposure or overexposure. During the illumination sensitivity analysis, only the illumination intensity was varied, whereas all other imaging parameters, including the camera position, exposure settings, imaging distance, and background, were kept unchanged.

The proposed method achieved the highest superpixel classification accuracy (90.79%) under the normal illumination condition. As the illumination deviated from the optimal level, the classification accuracy gradually decreased, reaching 77.18% under very dark conditions and 78.54% under very bright conditions. This trend indicates that both underexposure and overexposure reduce the discriminative power of color features.

Visual inspection of Figure 38 shows that low illumination mainly caused portions of the meat region to be misclassified as pickled side dish, whereas excessive illumination increased confusion between the egg and meat regions and between pickled side dish and rice. In contrast, the vegetable and rice regions remained relatively stable across different illumination levels. Overall, the proposed system maintained satisfactory classification performance under moderate illumination variations, demonstrating good adaptability for practical inspection environments.

To evaluate the feasibility of the proposed framework under varying illumination conditions, an independent image dataset was acquired using the digital LED light controller. Images were captured under seven illumination levels while maintaining the same imaging configuration and using the optimized parameters determined in Section 4.3. Therefore, the classification accuracies reported in Table 15 were obtained from this independently acquired dataset and should not be directly compared with the baseline performance reported in Table 5 and Table 12, which was evaluated using the testing dataset summarized in Table 3.

#### 4.6.3. Effect of Dynamic Conveyor-Based Imaging on Detection Performance

**Figure 39:**
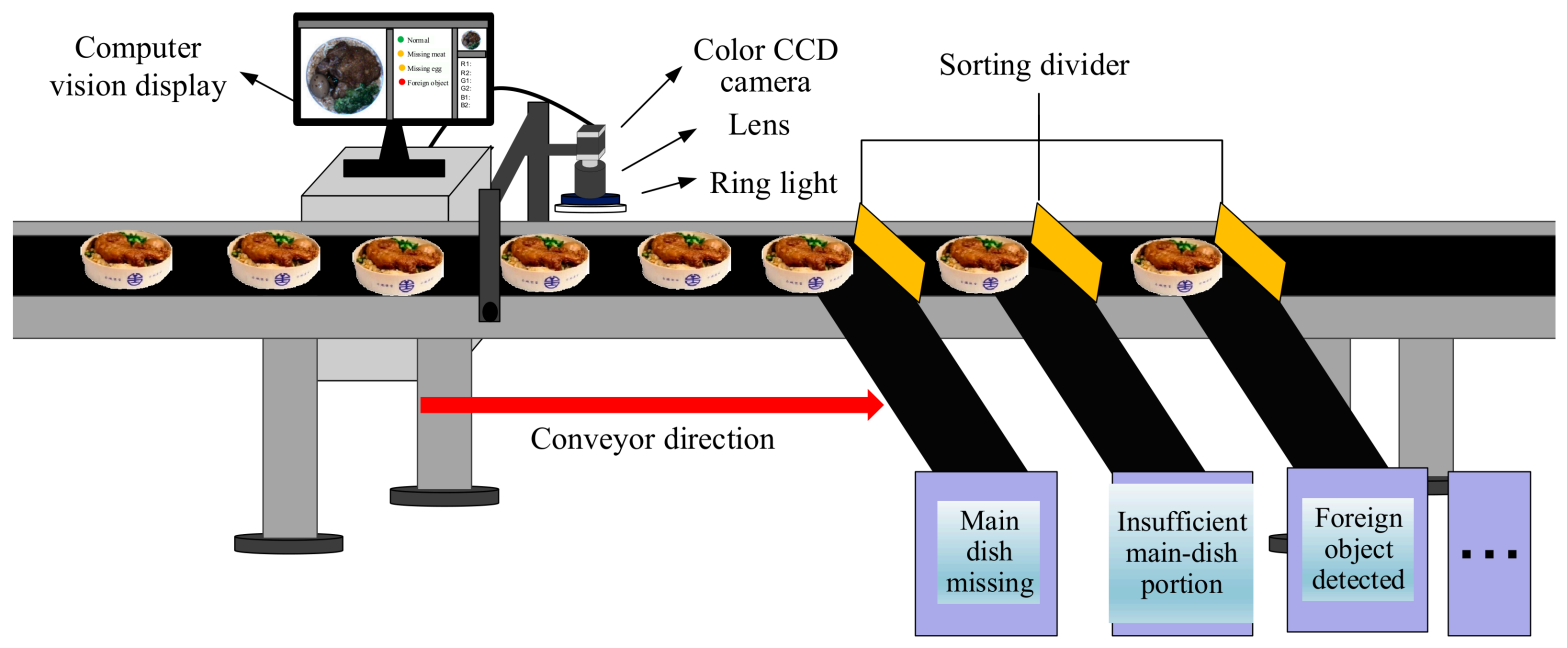
Conceptual diagram of the proposed conveyor-based lunch-box inspection system.

To evaluate the feasibility of the proposed system for real-time production, a conveyor-based inspection platform was developed for dynamic lunch-box inspection. As illustrated in Figure 39, lunch boxes were transported through an imaging station, where images were captured and analyzed for defect detection. Defective products were then directed to different sorting lanes according to the detected defect type.

**Figure 40:**
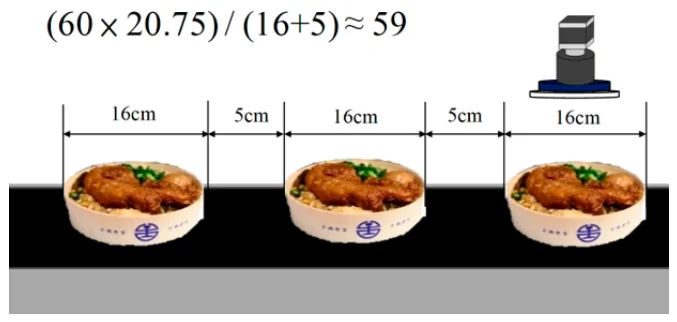
Example of inspection throughput calculation at conveyor speed setting 50.

The conveyor speed was varied to investigate the influence of motion blur on inspection performance. The corresponding conveyor speeds and inspection throughputs are summarized in Table 16. Inspection throughput was calculated using Equation (11), and an example is illustrated in Figure 40. Two experiments were conducted. In Stage I, food classification performance was evaluated at seven conveyor-speed settings. In Stage II, lunch boxes containing a simulated foreign object (cockroach) were inspected at three representative speeds (20, 50, and 70).

(11) $$E\approx \frac{60\times V_{Actual}}{D_{box}{+D}_{spacing}}$$

where *E*: inspection throughput (pieces/min), *V*: actual conveyor speed (cm/s), *Dbox*: lunch-box diameter (cm), and *Dspacing*: distance between adjacent lunch boxes (cm).

**Figure 41:**
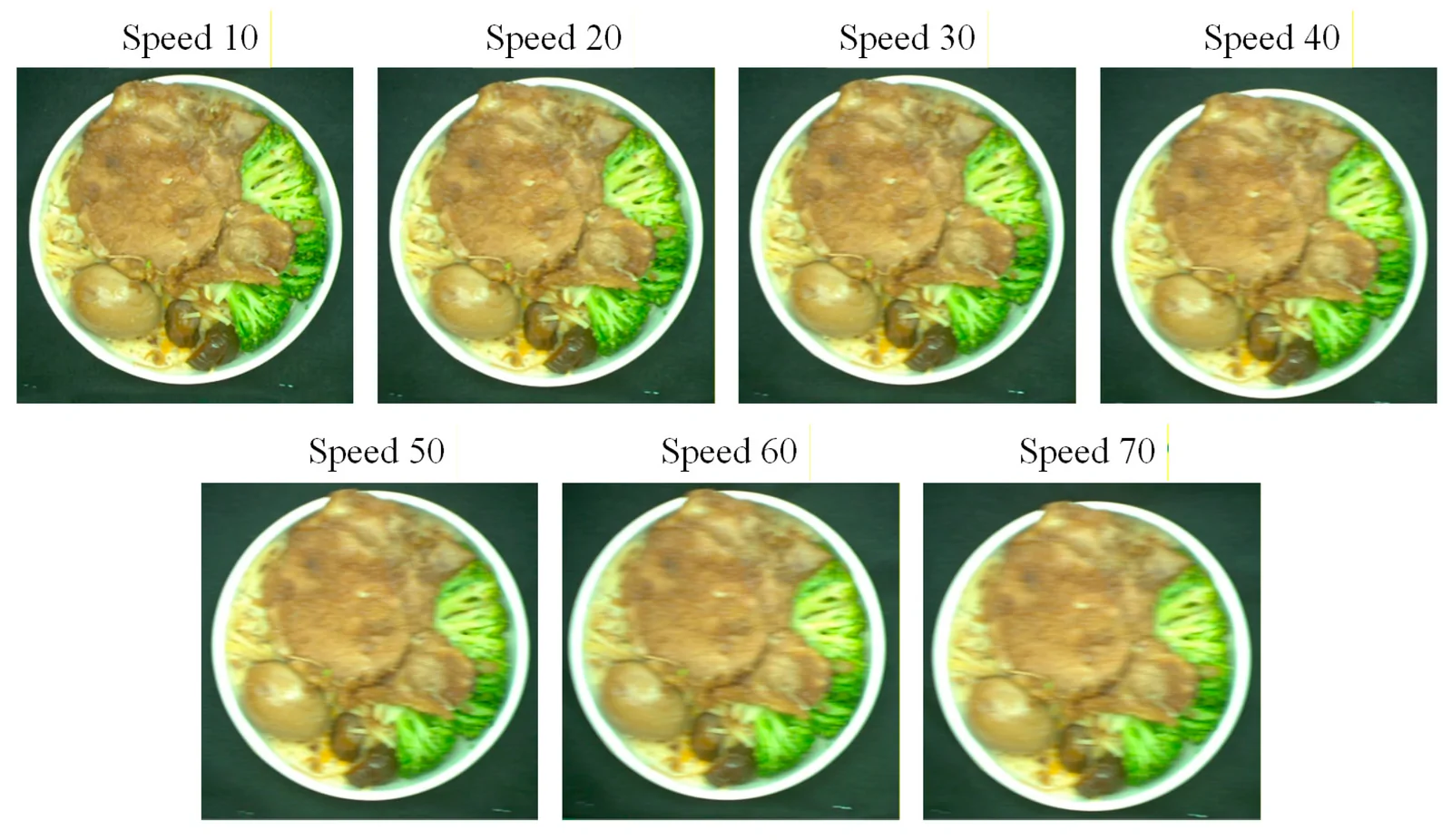
Representative lunch-box images captured at different conveyor speeds.

**Figure 42:**
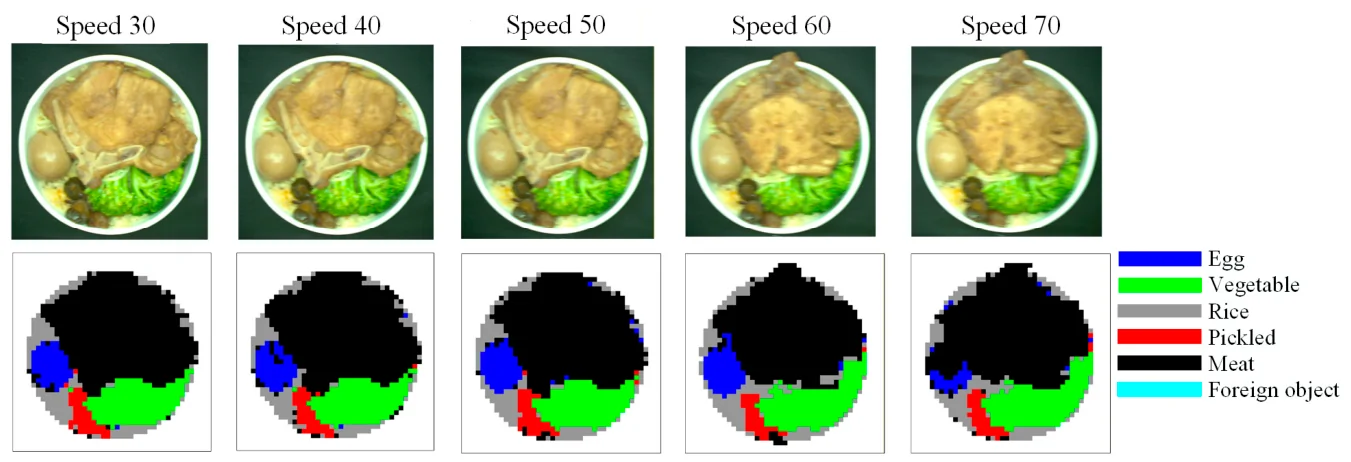
Food classification results under different conveyor speeds.

Representative images acquired at different conveyor speeds are shown in Figure 41. As the conveyor speed increased, motion blur became more pronounced, particularly along food boundaries. Nevertheless, the overall food appearance remained distinguishable. The quantitative results (Table 17) and representative classification results (Figure 42) show that superpixel classification accuracy decreased only slightly, from 91.76% at Speed 20 to 89.44% at Speed 70, demonstrating good adaptability for moderate motion blur.

**Figure 43:**
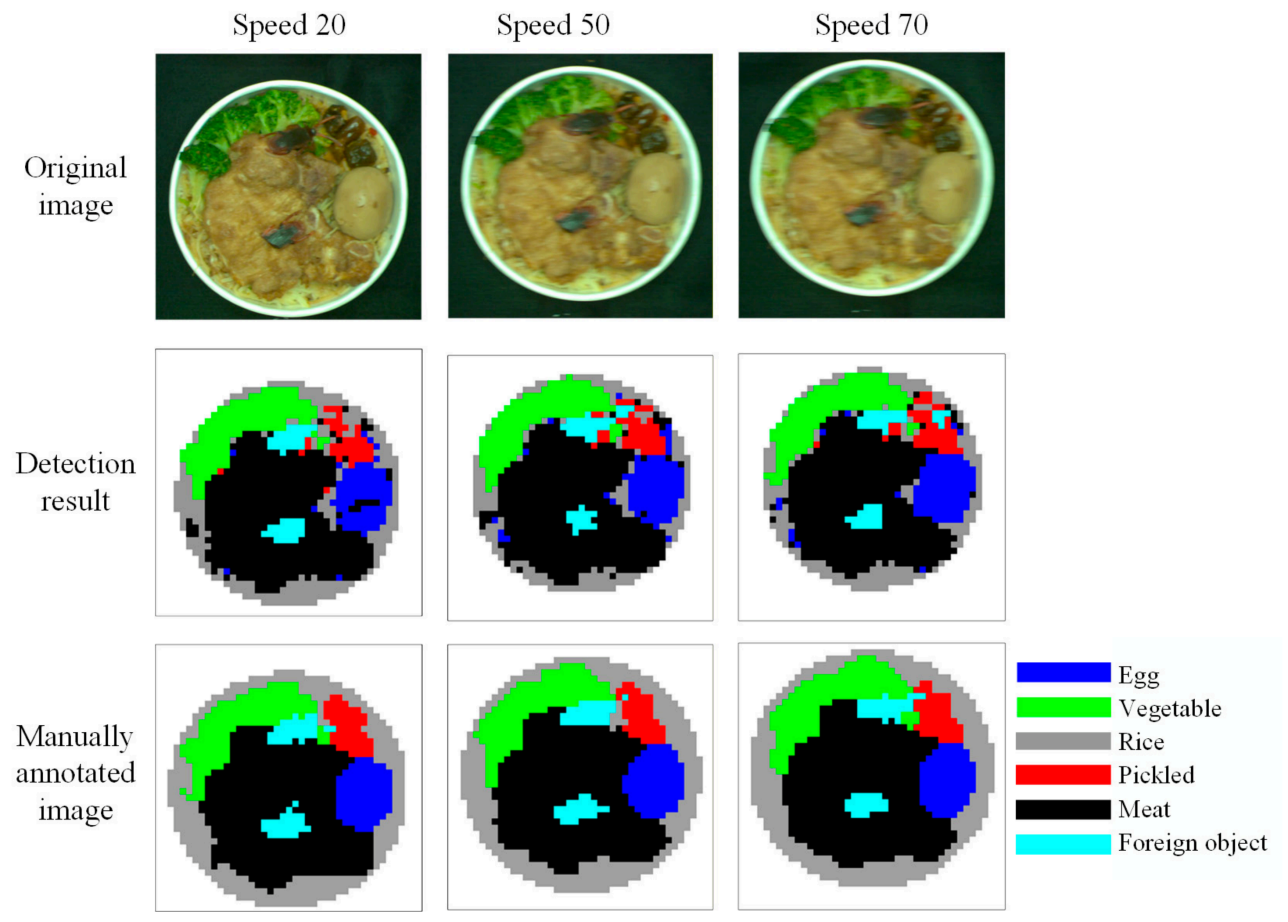
Foreign-object detection results under different conveyor speeds.

To further investigate the influence of conveyor speed on foreign-object detection, the Stage-II experiments evaluated representative low, medium, and high conveyor-speed settings. The corresponding quantitative results are summarized in Table 18, and representative detection results are shown in Figure 43. Foreign-object-containing samples were successfully identified at all three evaluated speeds; however, classification performance tended to deteriorate as conveyor speed increased because of increased motion blur. In particular, a higher normal-image false alarm rate was observed at the highest speed setting. Because the Stage-II experiment involved a limited number of samples, these percentage-based results are sensitive to individual classification errors and should be interpreted as preliminary rather than as statistically stable estimates of production-line performance.

Accordingly, the dynamic experiment should be regarded as a pilot feasibility evaluation of the proposed framework under conveyor-based image acquisition. Table 19 summarizes the relationship between conveyor speed and theoretical inspection throughput as a reference for the prototype system; it does not represent validated operating recommendations for industrial production. Establishing such recommendations would require substantially larger datasets collected at each conveyor speed, preferably across independent production batches and operating conditions, together with confidence-interval analysis of detection and false-positive rates. Nevertheless, the present results demonstrate the feasibility of applying the proposed framework to continuously moving lunch boxes and identify conveyor-induced motion blur as an important factor for subsequent large-scale validation.

### 4.7. Discussion and Limitations

The experimental results indicate that the effectiveness of the proposed framework arises from the complementary integration of geometric ROI extraction, fixed-grid regional representation, statistical color features, DNN classification, and flood-filling refinement. This combination provides a lightweight alternative to dense pixel-level segmentation by reducing computational complexity while retaining sufficient regional information for food recognition and quantitative defect analysis. Among the investigated parameters, a 6 × 6 grid provided the most favorable balance between spatial detail and computational efficiency. Although the proposed DNN outperformed SVM and Random Forest in the five-fold comparison, the contribution of this study should be interpreted primarily as an integrated lightweight inspection framework rather than a fundamentally new learning architecture.

Direct numerical comparisons with published meal-inspection systems should be made cautiously because existing studies differ substantially in datasets, food categories, acquisition conditions, annotation protocols, and evaluation metrics. Compared with dense semantic or instance segmentation approaches, the proposed framework emphasizes lower annotation and computational requirements through fixed-grid regional classification; however, controlled benchmarking against modern segmentation architectures using the same dataset and evaluation protocol is required to establish its relative performance. More rigorous multi-objective optimization, such as Pareto-based parameter selection [40], could further improve the trade-off among classification performance, computational cost, and processing time.

The sensitivity experiments demonstrated relatively stable performance under the evaluated variations in illumination, lunch-box geometry, food composition, and conveyor speed. However, these findings should be interpreted within the scope of the controlled laboratory and prototype conditions. Substantial illumination changes and increasing motion blur degraded classification performance, particularly for small or partially occluded food regions. Accordingly, the dynamic conveyor experiments should be regarded as a demonstration of technical feasibility rather than validation of industrial operating limits. Larger independent datasets, repeated acquisition sessions, and production-line trials are required to quantify performance variability and establish statistically reliable operating conditions.

Several methodological limitations should also be acknowledged. First, the classifier was developed for predefined TRA lunch-box categories and therefore requires additional data collection, annotation, and model retraining or adaptation when substantially different menu items are introduced. The empirical food-quantity thresholds must likewise be recalibrated when nominal portion sizes or presentation conditions change. The present study did not quantify the minimum amount of additional training data or the associated annotation and retraining costs required for menu adaptation; these maintenance requirements therefore remain to be investigated. Transfer learning, continual learning, and domain adaptation may help reduce the data and computational requirements associated with menu changes. Second, the fixed-grid color-based representation is more sensitive to small, partially occluded, visually similar, and boundary regions than to large and homogeneous food regions. Egg classification was particularly challenging because of partial occlusion and visual similarity with adjacent foods. Nevertheless, 23 of 25 missing-egg samples were correctly identified at the image level, indicating that localized superpixel-level errors do not necessarily propagate directly to the final defect decision. Third, the empirical food-quantity thresholds are menu-dependent and were calibrated using a limited number of defective samples; they should therefore not be interpreted as statistically universal decision limits. Future studies using larger datasets should investigate more rigorous threshold-selection strategies, including ROC-based optimization, Youden’s index, bootstrap analysis, and cost-sensitive decision criteria.

Foreign-object detection represents a particularly important limitation because of its direct relevance to food safety. The five-fold mean superpixel-level foreign-object detection rate was 82.12 ± 0.79%, and the evaluation was limited to a simulated plastic cockroach under controlled conditions. The present results should therefore be interpreted as proof-of-concept evidence rather than validation of a general foreign-object detection capability. Contaminants with different optical and physical characteristics, such as transparent plastic, reflective metal, hair, bone fragments, packaging materials, or objects visually similar to surrounding foods, may be considerably more difficult to detect using RGB color features alone. Furthermore, the 50% foreign-object detection criterion was empirically selected as an operational threshold and requires broader validation. Future experiments should therefore include contaminants with systematic variations in material, size, color, position, food background, and degree of occlusion, together with stricter evaluation of false-negative detection.

Future work should focus on broader external validation and methodological extension. Larger datasets acquired across independent production batches, meal types, operators, and environmental conditions are needed to evaluate generalizability beyond the present experimental setting. Direct numerical comparison with modern semantic or instance segmentation methods has not been experimentally established in the present study. Although the proposed framework emphasizes reduced annotation requirements and computational complexity through fixed-grid regional classification, controlled benchmarking against architectures such as U-Net, DeepLab, Mask R-CNN, and YOLO-based segmentation methods using identical datasets and evaluation protocols is required to determine their relative performance. The present experimental design evaluated individual defect categories separately and did not include lunch-box images containing multiple simultaneous abnormalities. Consequently, the current decision framework’s ability to identify and report multiple concurrent defects has not yet been validated and should be investigated in future multi-label inspection studies. Depth, multispectral, or hyperspectral imaging may further improve the discrimination of visually similar foods and challenging contaminants. Following broader validation, integration with edge-computing platforms and automated sorting equipment could be investigated toward real-time quality inspection in practical food-production environments.

## 5. Concluding Remarks

This study presents a vision-based framework for multi-object recognition and quantitative defect analysis in complex meal images. The proposed framework integrates geometric ROI extraction, fixed-grid regional feature representation, DNN classification, flood-filling refinement, and empirical food-quantity thresholds for automated quality inspection of boxed meals. It enables integrated food recognition and the detection of missing food items, insufficient portions, and foreign objects. Five-fold cross-validation further demonstrated stable classifier performance across different data partitions, with the proposed DNN achieving an overall image classification rate of 96.45 ± 1.08%.

Sensitivity experiments involving illumination variation, different lunch-box geometry and food composition, and conveyor-based image acquisition demonstrated the feasibility and stability of the framework under the evaluated laboratory and prototype conditions. However, these experiments do not constitute comprehensive validation across industrial environments. Performance may decrease for unseen meal configurations, severe food occlusion, substantial illumination changes, stronger motion blur, and small or visually similar food regions.

In addition, foreign-object detection was evaluated using only a simulated plastic cockroach and therefore requires substantially broader validation using contaminants with different materials, sizes, appearances, food backgrounds, and degrees of occlusion. Future work should also evaluate the proposed lightweight framework against modern semantic and instance segmentation methods using larger independently acquired datasets and actual production-line conditions. Such investigations are necessary to establish broader generalizability and suitability for industrial deployment.

## Figures and Tables

**Table 1:** Main parameters of the proposed DNN model.

Parameter	Setting
Input features	Food feature values: *μ_R_*, *μ_G_*, *μ_B_*, *σ_R_*, *σ_G_*, *σ_B_*, *μ_H_*, *μ_S_*, *μ_V_*, *σ_H_*, *σ_S_*, *σ_V_*, μ_L*_, μ_a*_, μ_b*_, σ_L*_, σ_a*_, σ_b*_, a total of 18 feature values.
*L* (Number of hidden layers)	*L* = 4
*N* (Number of nodes in each hidden layer)	*N* = 40
Output results	Classification outputs: *Y*_1_ (Meat), *Y*_2_ (Egg), *Y*_3_ (Vegetable), *Y*_4_ (Pickled side dish), *Y*_5_ (Rice), *Y*_6_ (Foreign object); a total of 6 output categories.

**Table 2:** Empirical food-quantity thresholds for detecting missing and insufficient food portions.

Food Category	*μ*	*σ*	Insufficient Portion Defect	Missing Food Defect
Meat	486.5	61.0	*μ* − 6*σ* < *X* ≤ *μ* − 2.2*σ*	*X* ≤ *μ* − 6*σ*
Egg	87.7	9.7	–	*X* ≤ *μ* − 5.9*σ*
Vegetable	204.1	48.3	*μ* − 3.2*σ* < *X* ≤ *μ* − 1.64*σ*	X ≤ *μ* − 3.2*σ*
Pickled side dish	57.0	17.7	–	*X* ≤ *μ* − 2.4*σ*

where *μ* = mean food area and *σ* = standard deviation of the food area obtained from the normal training samples; *X* = measured area of the food category in the test image. Note: Insufficient-portion thresholds were defined only for food categories whose serving quantity can be reliably evaluated by area. For discrete food items (e.g., eggs) and highly variable side dishes (e.g., pickled vegetables), only missing-food defects were considered.

**Table 3:** Summary of the image dataset used for training, validation, and testing. The training dataset was used for classifier learning and empirical food-quantity threshold determination; the validation dataset was used for parameter optimization; and the testing dataset was reserved for the final performance evaluation.

Dataset Type	Normal	Missing Meat	Missing Egg	Missing Vegetable	Insufficient Meat	Insufficient Vegetable	Missing Pickled Side Dish	Foreign Object	Total
Training images	200	10	10	10	10	10	10	50	310
Validation images	50	5	5	5	5	5	5	10	90
Testing images	100	27	25	30	20	20	20	25	267
Total images	350	42	40	45	35	35	35	85	667

Note: Foreign-object contamination was simulated using a plastic cockroach model.

**Table 4:** Circular Hough Transform parameter settings and quantitative ROI localization performance.

Parameter	Setting	Mean IoU
Minimum radius	*Min_R* = 100 pixels	-
Maximum radius	*Max_R* = 130 pixels	-
Accumulator threshold	*P*_1_ = 15, 20, 30, 40	0.4067, 0.5443, 0.8874, 0.9642.

**Table 5:** Performance comparison for different superpixel sizes.

Superpixel Size	5 × 5	6 × 6	7 × 7	8 × 8	9 × 9
Foreign-object detection rate (P_1−β)%	78.53	81.62	77.54	73.56	66.41
Food false-alarm rate (P_α)%	0.22	0.14	0.14	0.17	0.10
Food misclassification rate (P_γ)%	13.79	10.83	13.11	13.17	14.21
Superpixel classification accuracy (P_CR)%	86.21	89.17	86.89	86.83	85.79

**Table 6:** Architecture and fixed training parameters of the DNN classifier.

Category	Parameter	Value
Cost function	Loss function	Categorical cross-entropy
Optimizer	Optimization algorithm	Adam
Trainable parameters	Total trainable parameters	5926
Learning rate	Initial learning rate	0.001
Random seed	Random seed	42
Training epochs	Epochs	100
Stopping criterion	Training termination	Fixed at 100 epochs; no early stopping
Batch size	Batch size	200
Hidden layers	Activation function	ReLU
Hidden layers	Weight initializer	Normal
Output layer	Activation function	Softmax
Output layer	Weight initializer	Normal

**Table 7:** Parameter settings for the first-stage DNN architecture optimization.

Item	Parameter Setting
Input Features	*μ_R_*, *μ_G_*, *μ_B_*, *σ_R_*, *σ_G_*, *σ_B_*, *μ_H_*, *μ_S_*, *μ_V_*, *σ_H_*, *σ_S_*, *σ_V_*, μ_L*_, μ_a*_, μ_b*_, σ_L*_, σ_a__*_, σ_b__*_ (18 features)
*L* (Number of Hidden Layers)	1, 2, 3, 4, 5, 6
*N* (Number of Hidden Neurons per Layer)	20, 30, 40, 50, 60
Output Classes	*Y*_1_: Meat, *Y*_2_: Egg, *Y*_3_: Vegetable, *Y*_4_: Pickle, *Y*_5_: Rice, *Y*_6_: Foreign Object

**Table 8:** Performance comparison of different DNN architectures in the first-stage optimization. (*L*: number of hidden layers; *N*: number of hidden neurons per hidden layer).

	*N*	20	30	40	50	60
*L*						
1		(1−β)%: 91.01 α%: 9.00 γ%: 4.79 CR%: 91.01	(1−β)%: 86.22 α%: 16.00 γ%: 10.77 CR%: 85.39	(1−β)%: 87.42 α%: 19.00 γ%: 8.98 CR%: 85.01	(1−β)%: 93.41 α%: 19.00 γ%: 2.99 CR%: 88.76	(1−β)%: 89.82 α%: 20.00 γ%: 7.18 CR%: 86.14
2		(1−β)%: 92.21 α%: 18.00 γ%: 5.98 CR%: 88.39	(1−β)%: 83.23 α%: 26.00 γ%: 14.37 CR%: 79.77	(1−β)%: 86.22 α%: 23.00 γ%: 10.77 CR%: 82.77	(1−β)%: 89.12 α%: 14.00 γ%: 7.78 CR%: 88.01	(1−β)%: 85.02 α%: 16.00 γ%: 13.17 CR%: 84.46
3		(1−β)%: 86.82 α%: 35.00 γ%: 7.78 CR%: 78.65	(1−β)%: 80.23 α%: 17.00 γ%: 18.50 CR%: 81.27	(1−β)%: 85.02 α%: 23.00 γ%: 9.58 CR%: 82.02	(1−β)%: 83.23 α%: 20.00 γ%: 15.50 CR%: 82.02	(1−β)%: 82.23 α%: 12.00 γ%: 7.98 CR%: 83.42
4		(1−β)%: 96.96 α%: 8.00 γ%: 1.21 CR%: 95.47	(1−β)%: 97.57 α%: 7.00 γ%: 1.21 CR%: 95.84	(1−β)%: 98.20 α%: 5.00 γ%: 0.59 CR%: 97	(1−β)%: 95.15 α%: 7.00 γ%: 1.20 CR%: 94.33	(1−β)%: 94.54 α%: 6.00 γ%: 1.21 CR%: 94.33
5		(1−β)%: 96.40 α%: 10.00 γ%: 1.20 CR%: 94.01	(1−β)%: 95.20 α%: 10.00 γ%: 1.20 CR%: 93.25	(1−β)%: 95.8 α%: 12.00 γ%: 1.20 CR%: 92.88	(1−β)%: 95.20 α%: 11.00 γ%: 0.59 CR%: 92.88	(1−β)%: 94.61 α%: 10.00 γ%: 1.19 CR%: 92.88
6		(1−β)%: 92.81 α%: 8.00 γ%: 2.99 CR%: 92.50	(1−β)%: 92.28 α%: 9.00 γ%: 4.19 CR%: 92.13	(1−β)%: 92.81 α%: 8.00 γ%: 5.38 CR%: 92.50	(1−β)%: 94.01 α%: 8.00 γ%: 1.19 CR%: 93.25	(1−β)%: 90.41 α%: 12.00 γ%: 6.58 CR%: 89.51

**Table 9:** Performance comparison of different hidden-neuron configurations in the second-stage DNN architecture optimization (*L* = 4).

	*N*	30	35	40	45	50
*L*						
4		(1−β)%: 97.57 α%: 7.00 γ%: 1.21 CR%: 95.84	(1−β)%: 95.15 α%: 8.00 γ%: 0.60 CR%: 93.39	(1−β)%: 98.20 α%: 5.00 γ%: 0.60 CR%: 97	(1−β)%: 93.93 α%: 8.00 γ%: 0.61 CR%: 93.21	(1−β)%: 95.15 α%: 7.00 γ%: 1.20 CR%: 94.33

**Table 10:** Training dataset for empirical decision threshold determination.

Training Samples	Normal Images	Main Dish Omission (Meat)	Main Dish Omission (Egg)	Main Dish Omission (Vegetable)	Insufficient Portion (Meat)	Insufficient Portion (Vegetable)	Side Dish Omission (Pickle)	Total
Number of images	200	10	10	10	10	10	10	260
Threshold		NM	NE	NV	LM	LV	NP	

**Table 11:** Performance comparison of fixed and category-specific threshold strategies for food-defect detection.

Performance Metric	Parameter Group 1	Parameter Group 2	Parameter Group 3	Proposed Method
Insufficient-portion threshold	−3.0σ	−2.25σ	−1.5σ	LM = −2.2σ LV = −1.6σ
Food omission threshold	−6.0σ	−4.5σ	−3.0σ	NM = −6.0σ NE = −5.9σ NV = −3.2σ NP = −2.4σ
Defective-image detection rate (1−β) %	54.49	56.89	83.23	98.20
Normal-image false alarm rate α %	3.00	4.00	8.00	5.00
Defective-image misclassification rate γ %	18.56	21.56	10.18	0.60
Overall image classification rate CR %	70.41	71.54	86.52	97.00

**Table 12:** Performance comparison of SVM, Random Forest, and the proposed DNN.

Performance Metric	SVM	Random Forest	Proposed DNN
Optimal parameters	*C* = 16, *gamma* = 16	*n* = 20, *d* = 10	*L* = 4, *N* = 40
Foreign-object detection rate (P_1−β) %	79.13 ± 2.24	77.81 ± 2.82	82.25 ± 0.81
Food false detection rate (P_α) %	0.044 ± 0.033	0.108 ± 0.081	0.068 ± 0.032
Food misclassification rate (P_γ) %	10.88 ± 1.14	12.65 ± 1.43	10.03 ± 0.95
Superpixel classification accuracy (P_CR) %	87.83 ± 1.17	86.05 ± 1.35	89.46 ± 0.88
Defective-image detection rate (1−β) %	91.83 ± 2.25	89.34 ± 2.64	97.76 ± 1.21
Normal-image false alarm rate α %	7.33 ± 2.45	17.81 ± 3.87	5.34 ± 1.73
Defective-image misclassification rate γ %	5.43 ± 1.84	5.83 ± 2.03	0.82 ± 0.24
Overall image classification rate CR %	90.25 ± 1.72	86.82 ± 2.26	96.45 ± 1.08
Processing time (s/image)	0.790 ± 0.026	0.116 ± 0.008	0.084 ± 0.006

**Table 13:** Class-wise superpixel classification accuracy across five validation folds.

Class	Fold 1 (%)	Fold 2 (%)	Fold 3 (%)	Fold 4 (%)	Fold 5 (%)	Mean ± SD
Vegetable	93.2	92.5	94.0	92.8	93.6	93.22 ± 0.60
Meat	91.8	90.7	92.3	91.2	91.6	91.52 ± 0.61
Egg	79.4	77.8	80.1	78.5	79.0	78.96 ± 0.87
Pickled side dish	75.2	73.6	76.0	74.1	75.0	74.78 ± 0.94
Rice	90.4	89.1	90.0	89.5	90.2	89.84 ± 0.53
Foreign object	82.0	81.1	83.2	81.8	82.5	82.12 ± 0.79

**Table 14:** Confusion matrix for food classification of the TRA octagonal pork chop lunch box.

Ground Truth	Vegetable	Pork	Egg	Pickle	Rice	Fish	Total	Accuracy
Vegetable	846	6	0	4	46	0	902	93.80%
Pork	7	3049	22	16	64	21	3179	95.91%
Egg	0	98	374	3	53	1	529	70.69%
Pickle	2	8	4	232	41	2	289	80.27%
Rice	16	57	38	43	2142	1	2297	93.25%
Fish	5	55	16	26	80	561	743	75.50%
Total	876	3273	454	324	2426	586	7939	

**Table 15:** Classification performance evaluated using an independently acquired image dataset under different controlled illumination conditions.

Illumination Level	Very Dark	Moderately Dark	Slightly Dark	Normal Illumination	Slightly Bright	Moderately Bright	Very Bright
Controller setting	5	10	15	20	25	30	35
Mean brightness	71	72	78	94	107	124	140
Standard deviation	21	21	21	24	27	30	34
Superpixel classification accuracy, P_CR (%)	77.18	79.05	85.39	90.79	85.41	79.83	78.54

**Table 16:** Inspection throughput under different conveyor speed settings.

Conveyor Speed Setting	Speed 10	Speed 20	Speed 30	Speed 40	Speed 50	Speed 60	Speed 70
Actual speed (cm/s)	3.49	7.92	11.17	14.80	20.75	25.85	28.77
Inspection throughput (pieces/min)	10	23	32	42	59	74	82

**Table 17:** Stage-I food classification performance under different conveyor speeds.

Conveyor Speed	Speed 10	Speed 20	Speed 30	Speed 40	Speed 50	Speed 60	Speed 70
Vegetable accuracy (%)	96.99	95.04	94.70	95.82	94.22	92.47	92.80
Meat accuracy (%)	95.89	97.50	97.25	96.13	95.54	95.91	95.62
Egg Accuracy (%)	67.05	70.71	71.43	67.76	69.09	69.09	57.78
Pickle accuracy (%)	81.90	75.35	76.50	75.91	72.35	73.30	72.69
Rice accuracy (%)	85.42	85.46	84.06	82.90	86.74	83.22	85.49
Superpixel classification accuracy, P_CR (%)	91.47	91.76	91.52	90.69	90.95	90.09	89.44

**Table 18:** Stage-II foreign-object detection performance under different conveyor speeds.

Speed Setting	Speed 20	Speed 50	Speed 70
Foreign object detection rate, (P_1−β)%	67.03	58.02	56.24
Food false alarm rate, (P_α)%	0.16	0.19	0.19
Food misclassification rate, (P_γ)%	11.63	12.87	12.85
Superpixel classification accuracy, (P_CR)%	88.81	87.77	87.81
Defective image detection rate, (1−*β*)%	100	100	100
Normal image false positive rate, α%	0	0	20
Defective image misclassification rate, γ%	0	0	0
Overall image classification rate, CR%	100	100	95

**Table 19:** Relationship between conveyor speed setting and estimated inspection throughput for the prototype system.

Production Throughput (Pieces/Min)	0~20	21~30	31~40	41~50	51~70	71~80
Conveyor Speed Setting	20	30	40	50	60	70

## Data Availability

The complete image dataset, annotations, trained model weights, and source code generated or used in this study are not publicly available because of project-related data and implementation constraints. To support reproducibility, detailed descriptions of the dataset composition and partitioning, image acquisition and annotation procedures, feature extraction, model architecture, training configuration, parameter-selection procedures, and evaluation protocol are provided in the manuscript.

## References

[1] Brosnan T., Sun D.W. (2002). Inspection and grading of agricultural and food products by computer vision systems-a review. Comput. Electron. Agric..

[2] Brosnan T., Sun D.-W. (2004). Improving quality inspection of food products by computer vision-A review. J. Food Eng..

[3] Patel K.K., Kar A., Jha S.N., Khan M.A. (2012). Machine vision system: A tool for quality inspection of food and agricultural products. J. Food Sci. Technol..

[4] Sun D.-W. (2008). Computer Vision Technology for Food Quality Evaluation.

[5] Wu D., Sun D.W. (2013). Colour measurements by computer vision for food quality control-A review. Trends Food Sci. Technol..

[6] Zhou L., Zhang C., Liu F., Qiu Z., He Y. (2019). Application of deep learning in food: A review. Compr. Rev. Food Sci. Food Saf..

[7] Zhu L., Spachos P., Pensini E., Plataniotis K.N. (2021). Deep learning and machine vision for food processing: A survey. Curr. Res. Food Sci..

[8] Chhetri K.B. (2024). Applications of artificial intelligence and machine learning in food quality control and safety assessment. Food Eng. Rev..

[9] Liu C., Cao Y., Luo Y., Chen G., Vokkarane V., Ma Y. (2016). Deepfood: Deep learning-based food image recognition for computer-aided dietary assessment. International Conference on Smart Homes and Health Telematics.

[10] Pandey P., Deepthi A., Mandal B., Puhan N.B. (2017). FoodNet: Recognizing foods using ensemble of deep networks. IEEE Signal Process. Lett..

[11] Tahir G.A., Loo C.K. (2021). A Comprehensive Survey of Image-Based Food Recognition and Volume Estimation Methods for Dietary Assessment. Healthcare.

[12] Mansouri M., Benabdellah Chaouni S., Andaloussi S.J., Ouchetto O. (2023). Deep learning for food image recognition and nutrition analysis: Towards chronic diseases monitoring-A systematic review. SN Comput. Sci..

[13] Niu C., Ying X., Pei G., Hu M., Zhai G. (2024). Review of the deep learning for food image processing. Int. J. Agric. Biol. Eng..

[14] Farinella G.M., Allegra D., Moltisanti M., Stanco F., Battiato S. (2016). Retrieval and classification of food images. Comput. Biol. Med..

[15] Giovany S., Putra A., Hariawan A.S., Wulandhari L.A. (2017). Machine learning and SIFT approach for Indonesian food image recognition. Procedia Comput. Sci..

[16] Oliveira L., Costa V., Neves G., Oliveira T., Jorge E., Lizarraga M. (2014). A mobile, lightweight, poll-based food identification system. Pattern Recognit..

[17] Ciocca G., Napoletano P., Schettini R. (2016). Food recognition: A new dataset, experiments, and results. IEEE J. Biomed. Health Inform..

[18] Matsuda Y., Hoashi H., Yanai K. (2012). Recognition of multiple-food images by detecting candidate regions. Proceedings of the 2012 IEEE International Conference on Multimedia and Expo.

[19] Zhu F., Bosch M., Khanna N., Boushey C.J., Delp E.J. (2014). Multiple hypotheses image segmentation and classification with application to dietary assessment. IEEE J. Biomed. Health Inform..

[20] Chopra M., Purwar A. (2022). Food image recognition using CNN, faster R-CNN and YOLO. Applications of Artificial Intelligence, Big Data and Internet of Things in Sustainable Development.

[21] Sudharson S., Kokil P., Annamalai R., Manoj N.S. Enhanced food classification system using YOLO models for object detection algorithm. Proceedings of the 2023 14th International Conference on Computing Communication and Networking Technologies (ICCCNT).

[22] Tan Z., Dong G., Zhao C., Basu A., Berretti S., Azari H. (2025). Affine-Transformation-Invariant Image Classification by Differentiable Arithmetic Distribution Module. Smart Multimedia.

[23] Raju V.B., Sazonov E. (2020). A systematic review of sensor-based methodologies for food portion size estimation. IEEE Sens. J..

[24] Yang Y., Jia W., Bucher T., Zhang H., Sun M. (2019). Image-based food portion size estimation using a smartphone without a fiducial marker. Public Health Nutr..

[25] Vinod G., He J., Shao Z., Zhu F. Food portion estimation via 3D object scaling. Proceedings of the IEEE/CVF Conference on Computer Vision and Pattern Recognition.

[26] Fang S., Zhu F., Kerr D.A., Boushey C.J., Delp E.J. Single-view food portion estimation: Learning image-to-energy mappings using generative adversarial networks. Proceedings of the 2018 25th IEEE International Conference on Image Processing (ICIP).

[27] Raju V.B., Hossain D., Sazonov E. (2023). Estimation of plate and bowl dimensions for food portion size assessment in a wearable sensor system. IEEE Sens. J..

[28] Mohd Khairi M.T., Ibrahim S., Md Yunus M.A., Faramarzi M. (2018). Noninvasive techniques for detection of foreign bodies in food: A review. J. Food Process Eng..

[29] Yao J., Wu Z., Wang P., Ye J., Wang J. (2025). Research on X-ray Image Fusion Algorithm for Food Foreign Object Detection. J. Nondestruct. Eval..

[30] Rong D., Xie L., Ying Y. (2019). Computer vision detection of foreign objects in walnuts using deep learning. Comput. Electron. Agric..

[31] Son G.J., Kwak D.H., Park M.K., Kim Y.D., Jung H.C. (2021). U-Net-based foreign object detection method using effective image acquisition system: A case of almond and green onion flake food process. Sustainability.

[32] Xiao Z., Wang J., Han L., Guo S., Cui Q. (2022). Application of machine vision system in food detection. Front. Nutr..

[33] Lin Y., Ma J., Wang Q., Sun D.W. (2023). Applications of machine learning techniques for enhancing nondestructive food quality and safety detection. Crit. Rev. Food Sci. Nutr..

[34] Hemamalini V., Rajarajeswari S., Nachiyappan S., Sambath M., Devi T., Singh B.K., Raghuvanshi A. (2022). Food quality inspection and grading using efficient image segmentation and machine learning-based system. J. Food Qual..

[35] Canny J. (2009). A computational approach to edge detection. IEEE Trans. Pattern Anal. Mach. Intell..

[36] Duda R.O., Hart P.E. (1972). Use of the Hough transformation to detect lines and curves in pictures. Commun. ACM.

[37] Achanta R., Shaji A., Smith K., Lucchi A., Fua P., Süsstrunk S. (2012). SLIC superpixels compared to state-of-the-art superpixel methods. IEEE Trans. Pattern Anal. Mach. Intell..

[38] Gonzalez R.C., Woods R.E. (2018). Digital Image Processing.

[39] Heckbert P.S., Glassner A.S. (1990). Seed fill algorithms. Graphics Gems.

[40] Jin Y., Sendhoff B. (2008). Pareto-based multiobjective machine learning: An overview and case studies. IEEE Trans. Syst. Man Cybern. Part C (Appl. Rev.).

